# Supplementary material for: Impaired fatty acid import or catabolism in macrophages restricts intracellular growth of Mycobacterium tuberculosis
Source: eLife. 2025 Mar 13;13:RP102980. doi: 10.7554/eLife.102980 (PMC11906158; doi:10.7554/eLife.102980)

Figure 1 - figure supplement 1A

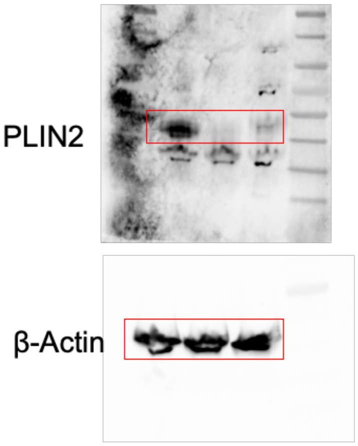

Figure 1 - figure supplement 1B

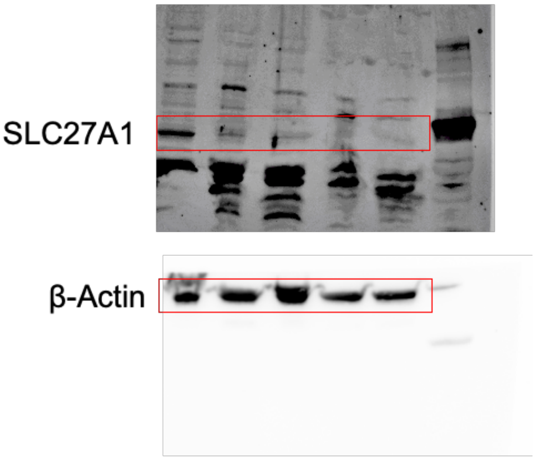

Figure 1 - figure supplement 1D

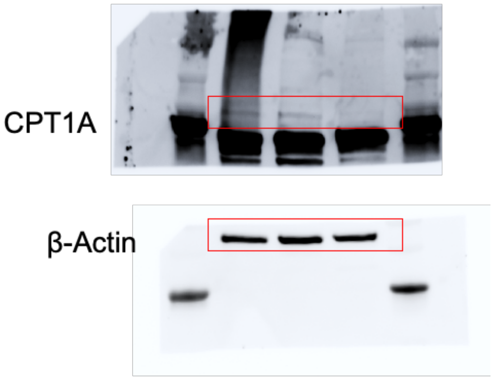

Figure 1 - figure supplement 1E

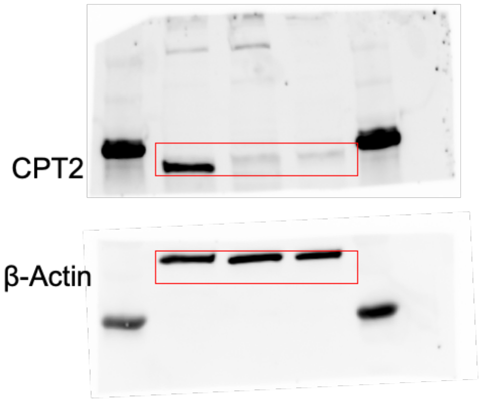

Supplement: Figure 1—figure supplement 1—source data 1. [file elife-102980-fig1-figsupp1-data1.pdf]
