# Supplementary figures and images for: Impaired fatty acid import or catabolism in macrophages restricts intracellular growth of Mycobacterium tuberculosis
Source: eLife. 2025 Mar 13;13:RP102980. doi: 10.7554/eLife.102980 (PMC11906158; doi:10.7554/eLife.102980)

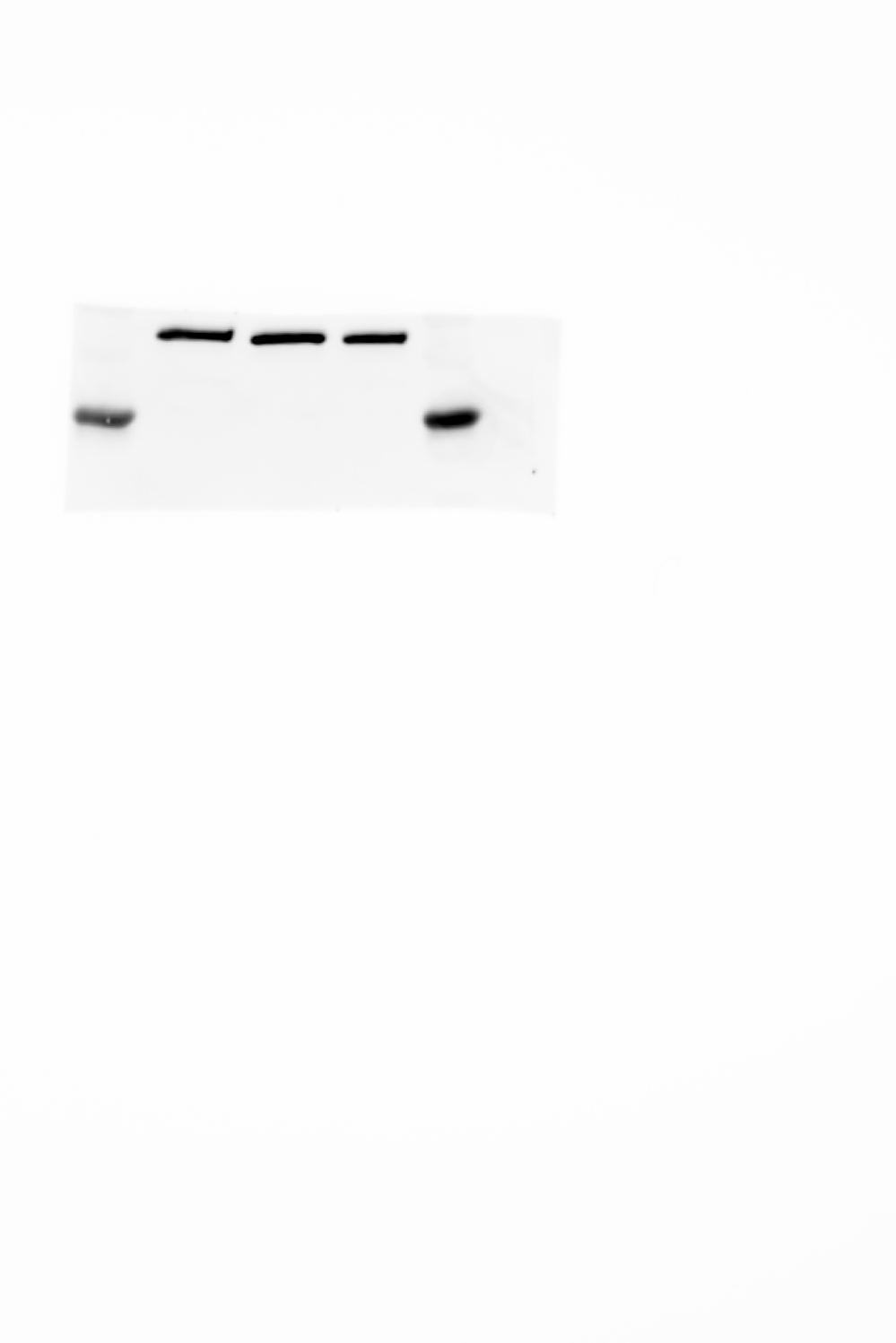

Supplement: Figure 1—figure supplement 1—source data 2. [file elife-102980-fig1-figsupp1-data2.zip › Figure 1 - figure supplement 1 - source data 2/bActin3_CPT2.tif]

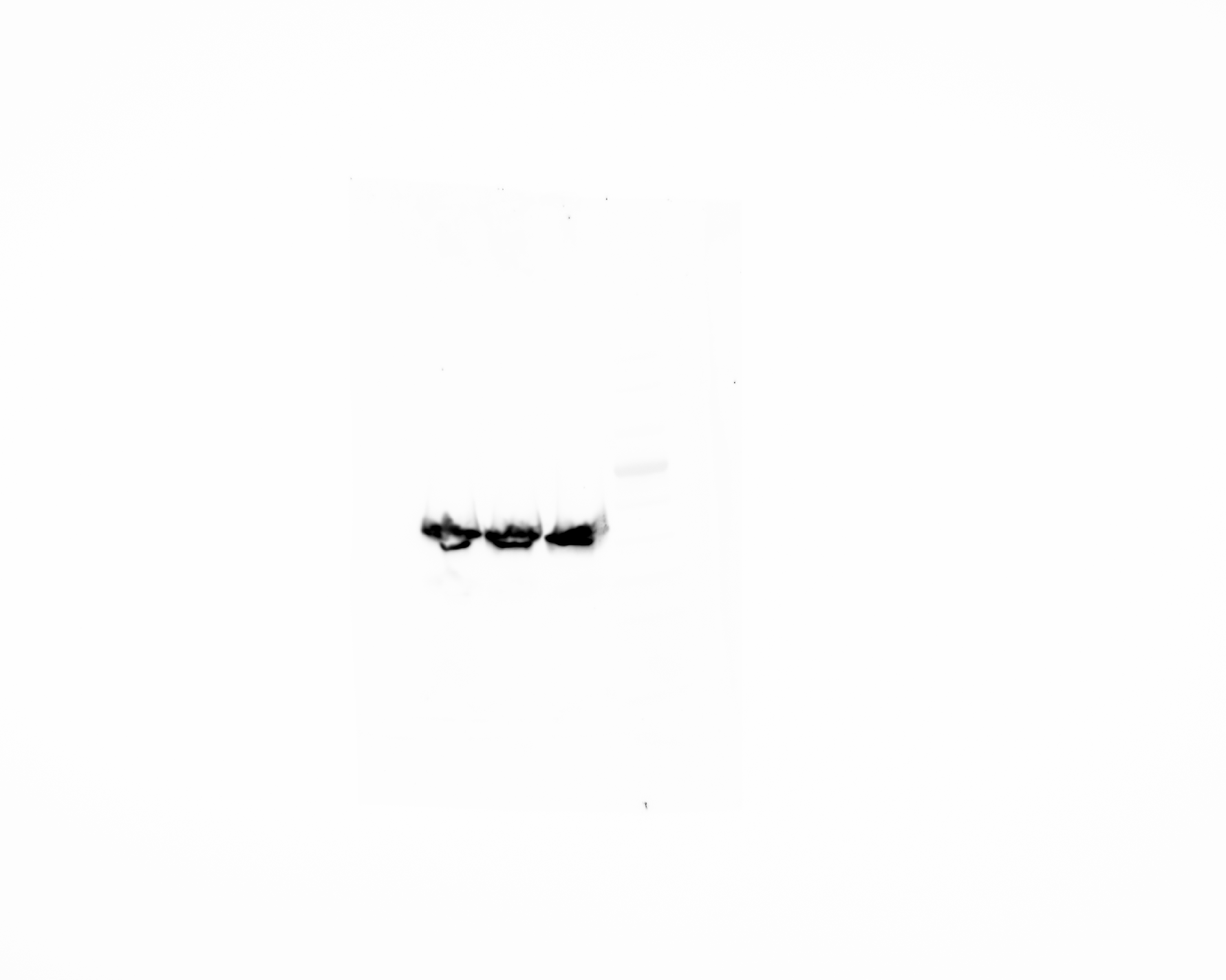

Supplement: Figure 1—figure supplement 1—source data 2. [file elife-102980-fig1-figsupp1-data2.zip › Figure 1 - figure supplement 1 - source data 2/bActin2_PLIN2.tif]

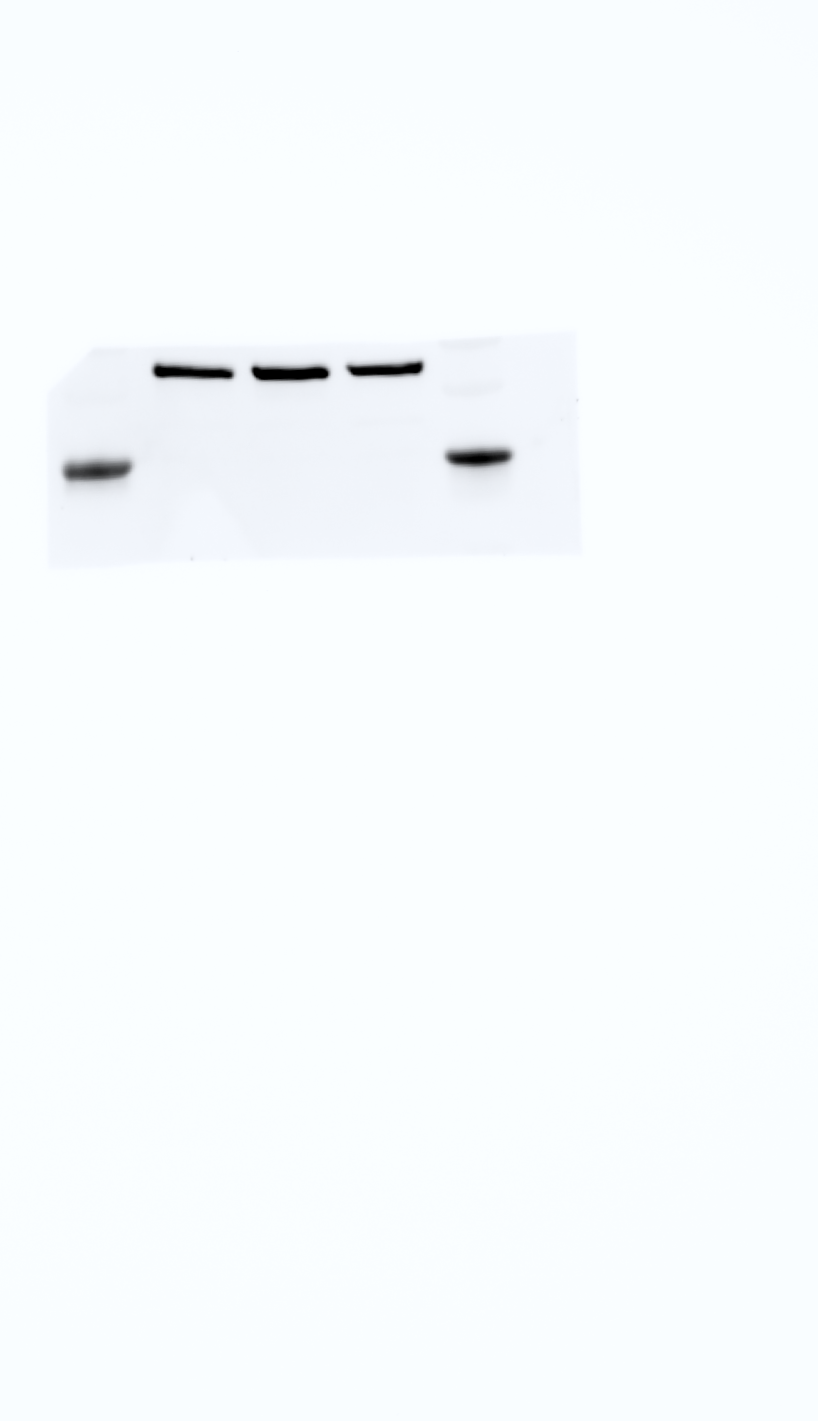

Supplement: Figure 1—figure supplement 1—source data 2. [file elife-102980-fig1-figsupp1-data2.zip › Figure 1 - figure supplement 1 - source data 2/bActin4_CPT1A.tif]

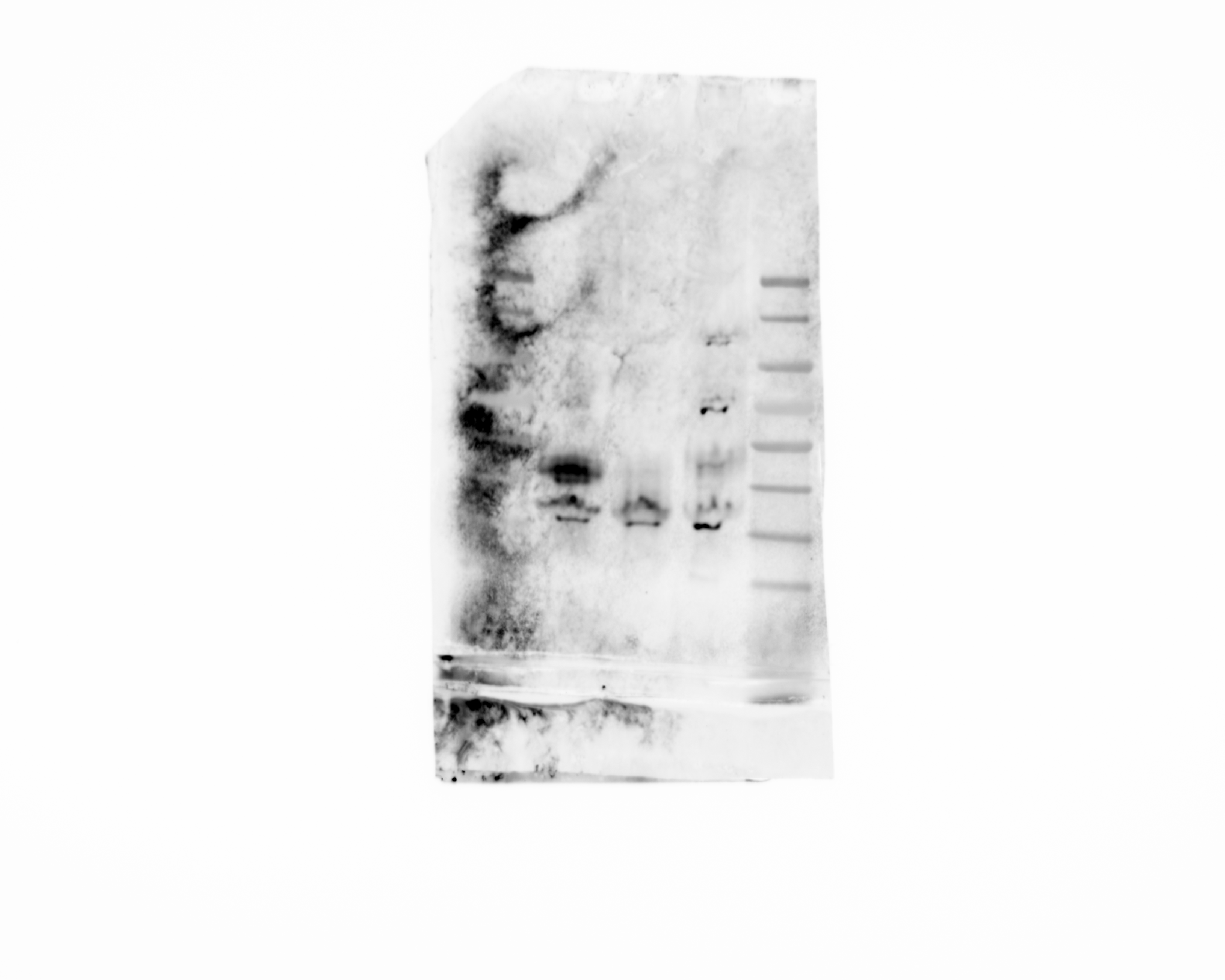

Supplement: Figure 1—figure supplement 1—source data 2. [file elife-102980-fig1-figsupp1-data2.zip › Figure 1 - figure supplement 1 - source data 2/PLIN2.tif]

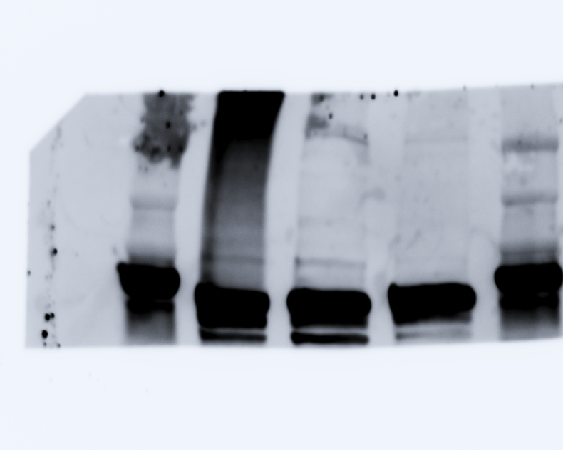

Supplement: Figure 1—figure supplement 1—source data 2. [file elife-102980-fig1-figsupp1-data2.zip › Figure 1 - figure supplement 1 - source data 2/CPT1A.tif]

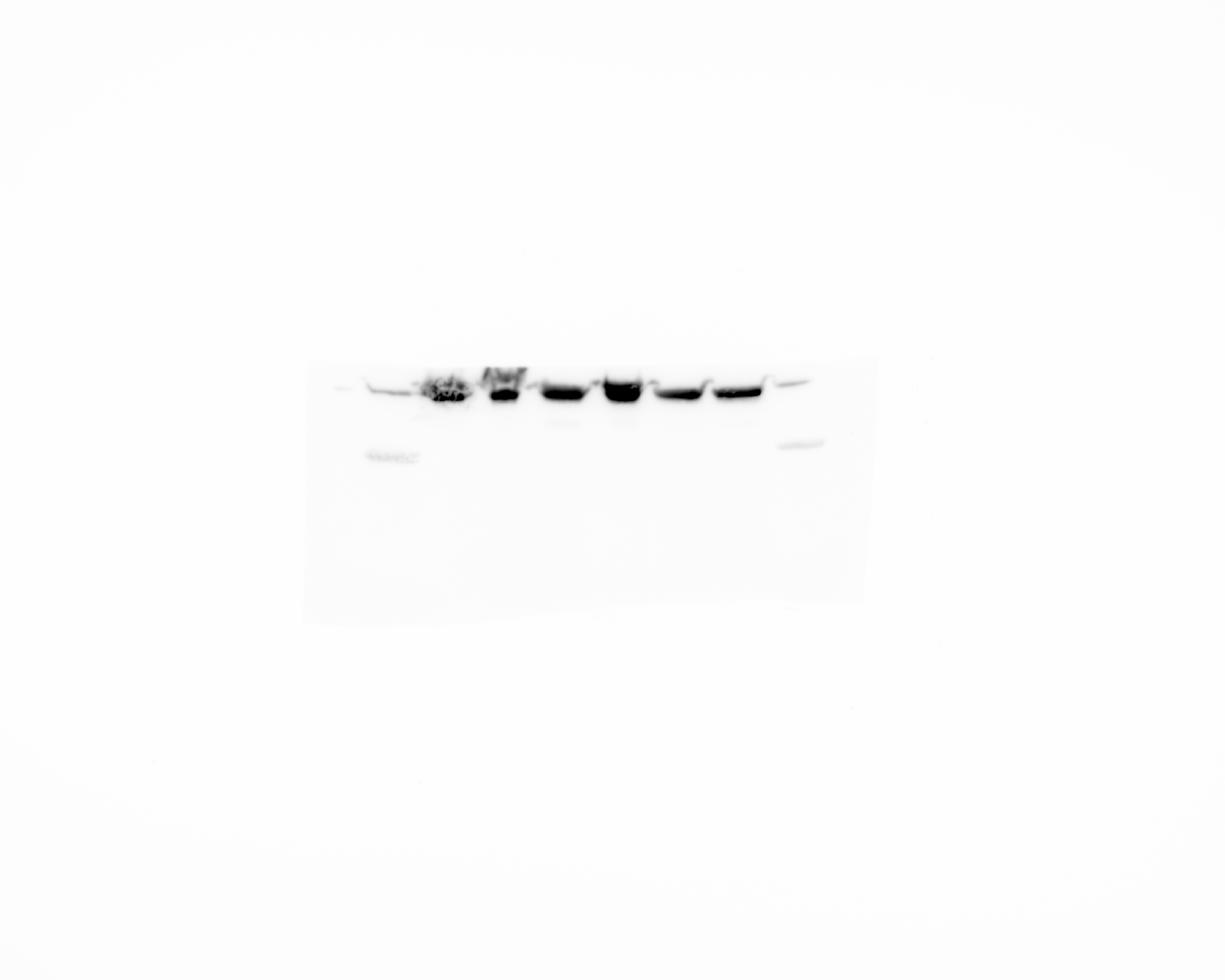

Supplement: Figure 1—figure supplement 1—source data 2. [file elife-102980-fig1-figsupp1-data2.zip › Figure 1 - figure supplement 1 - source data 2/bActin1_SLC27A1.tif]

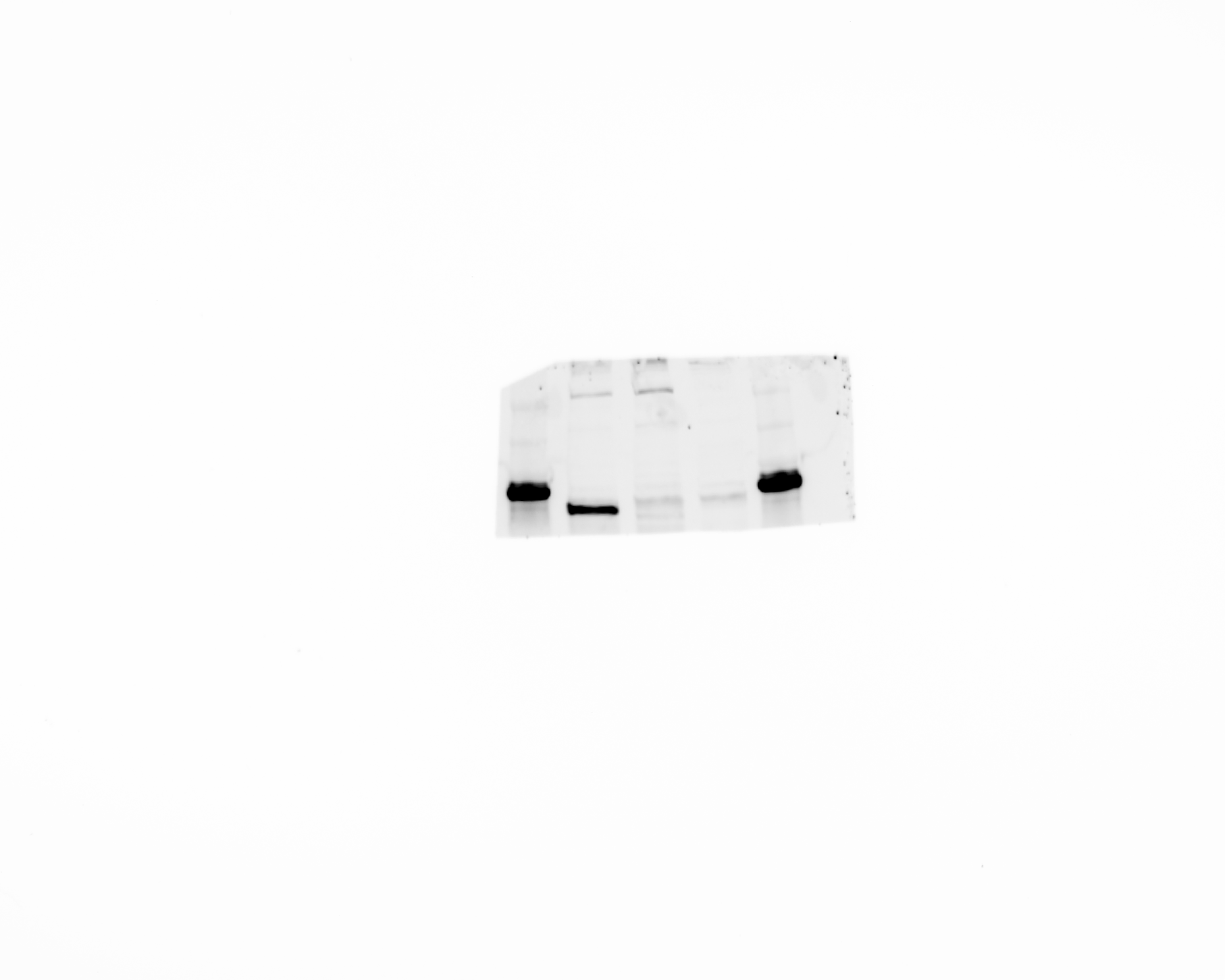

Supplement: Figure 1—figure supplement 1—source data 2. [file elife-102980-fig1-figsupp1-data2.zip › Figure 1 - figure supplement 1 - source data 2/CPT2.tif]

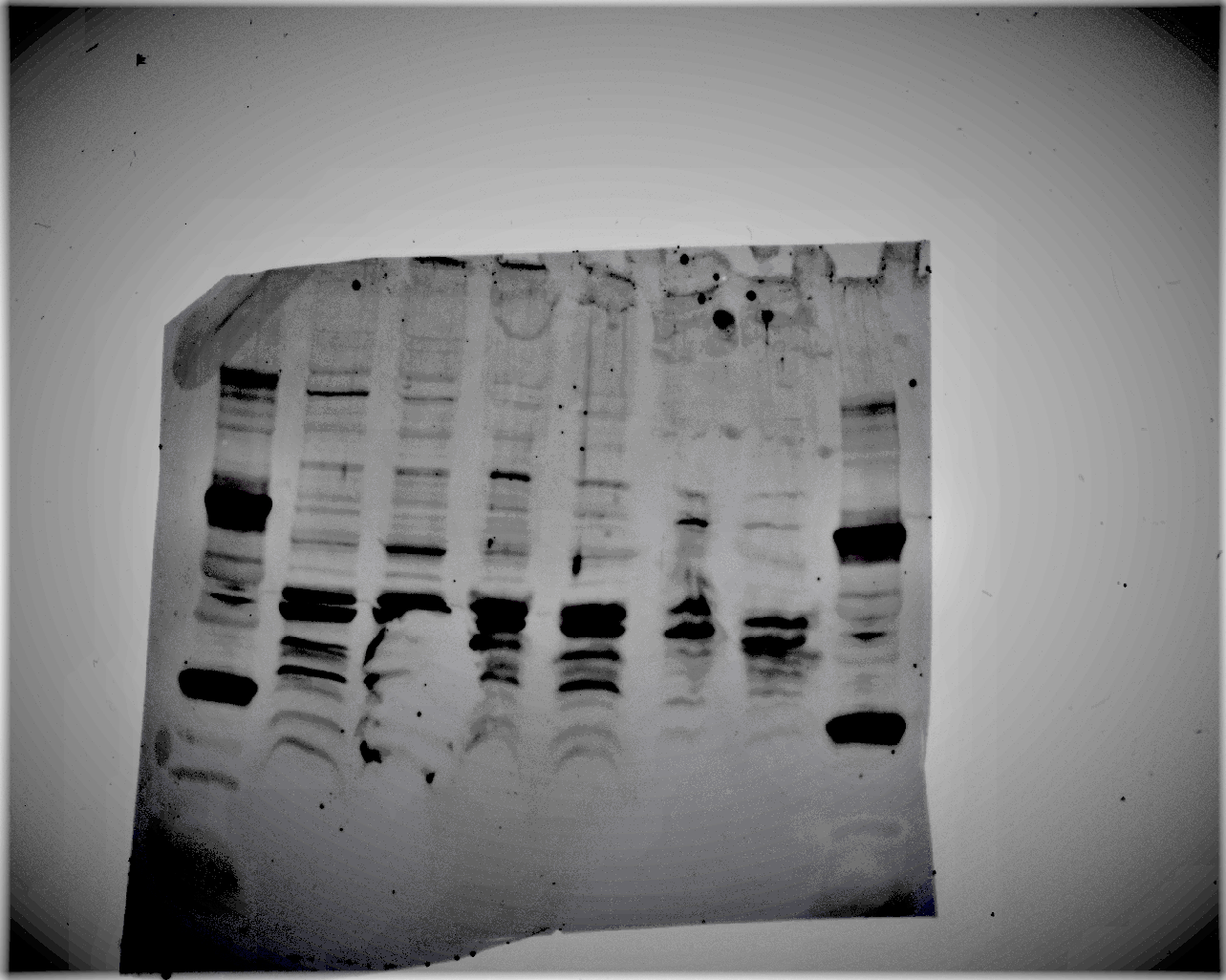

Supplement: Figure 1—figure supplement 1—source data 2. [file elife-102980-fig1-figsupp1-data2.zip › Figure 1 - figure supplement 1 - source data 2/SLC27A1.tif]

Figure 3A

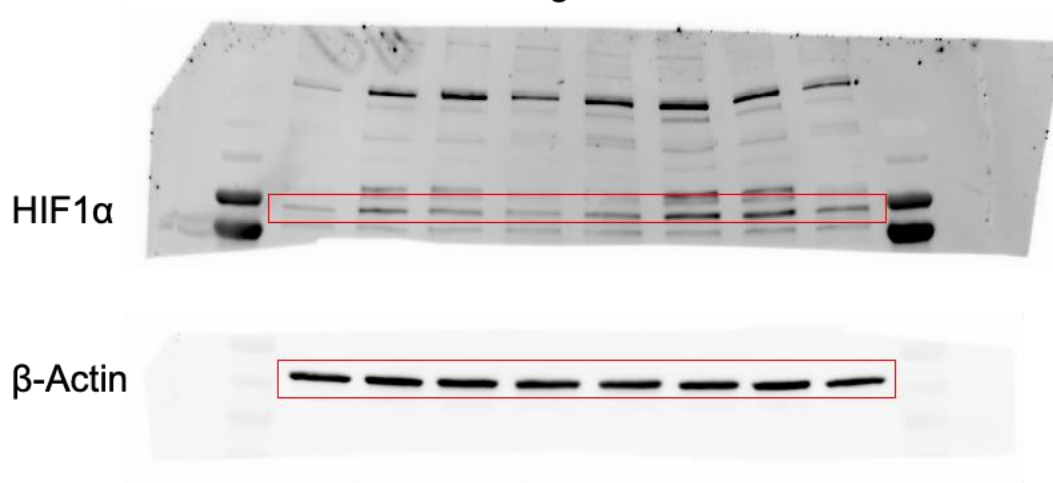

Figure 3C

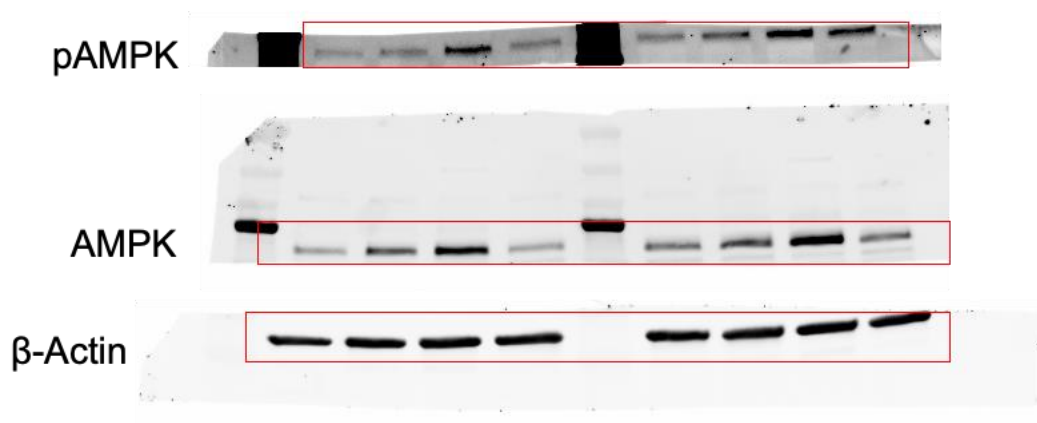

Supplement: Figure 3—source data 1. [file elife-102980-fig3-data1.pdf]

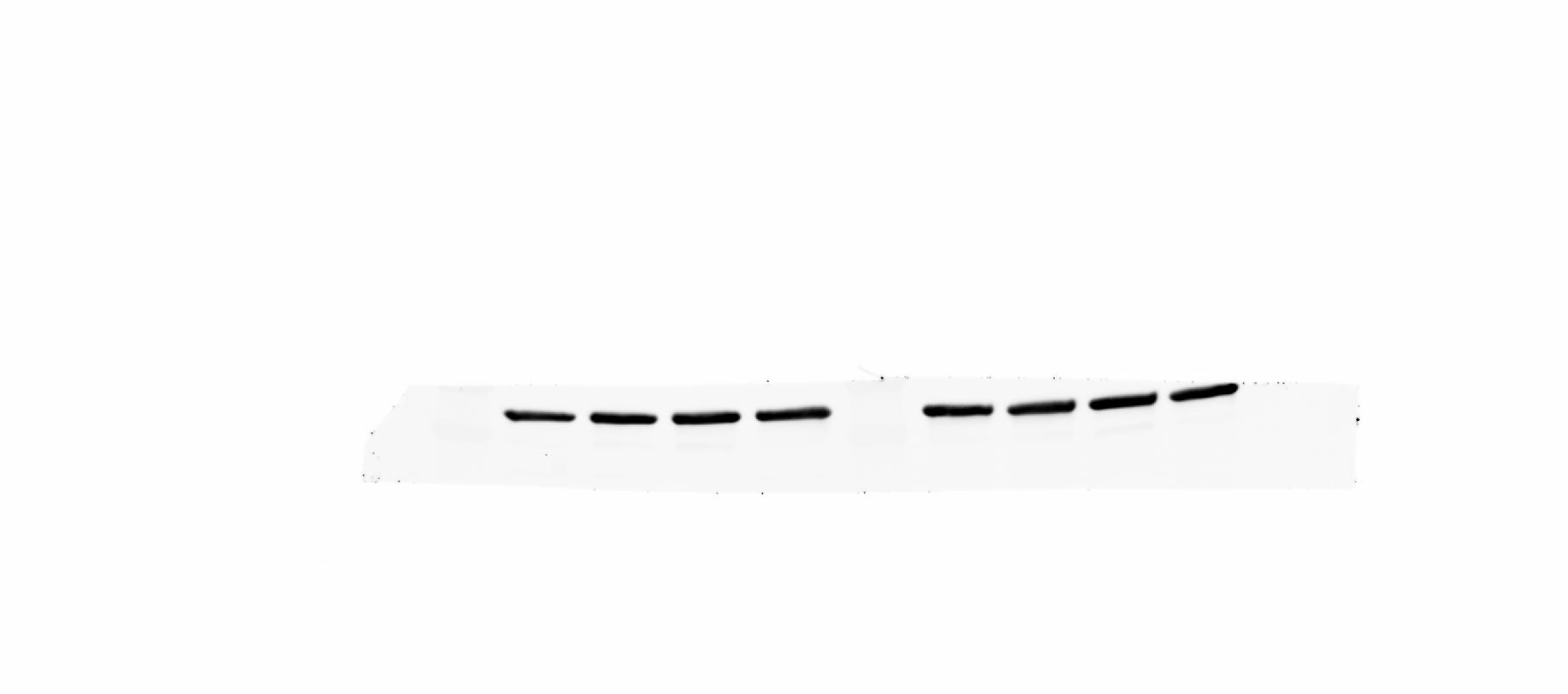

Supplement: Figure 3—source data 2. [file elife-102980-fig3-data2.zip › Figure 3 - source data 2/bActin2_AMPK.tif]

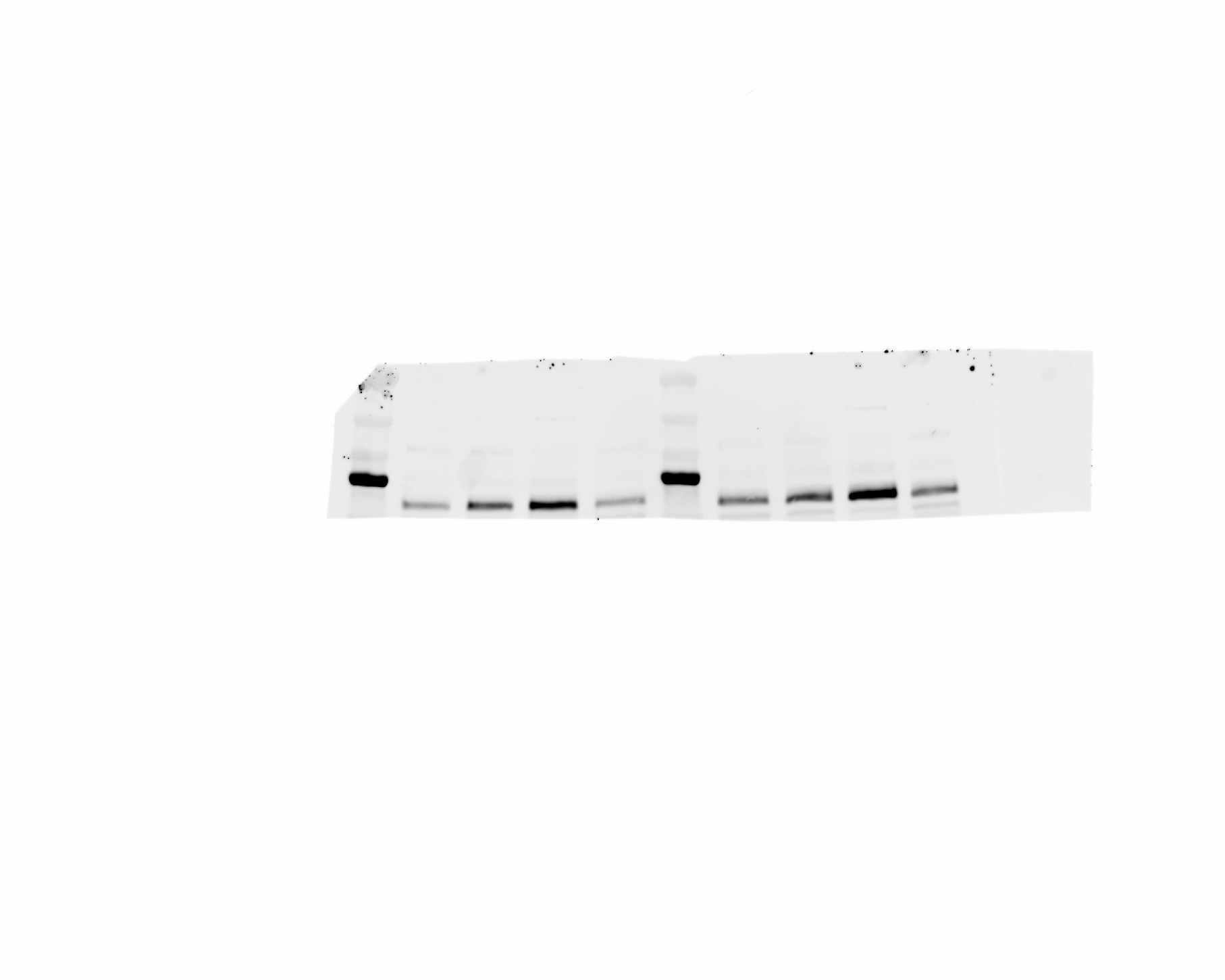

Supplement: Figure 3—source data 2. [file elife-102980-fig3-data2.zip › Figure 3 - source data 2/AMPK.tif]

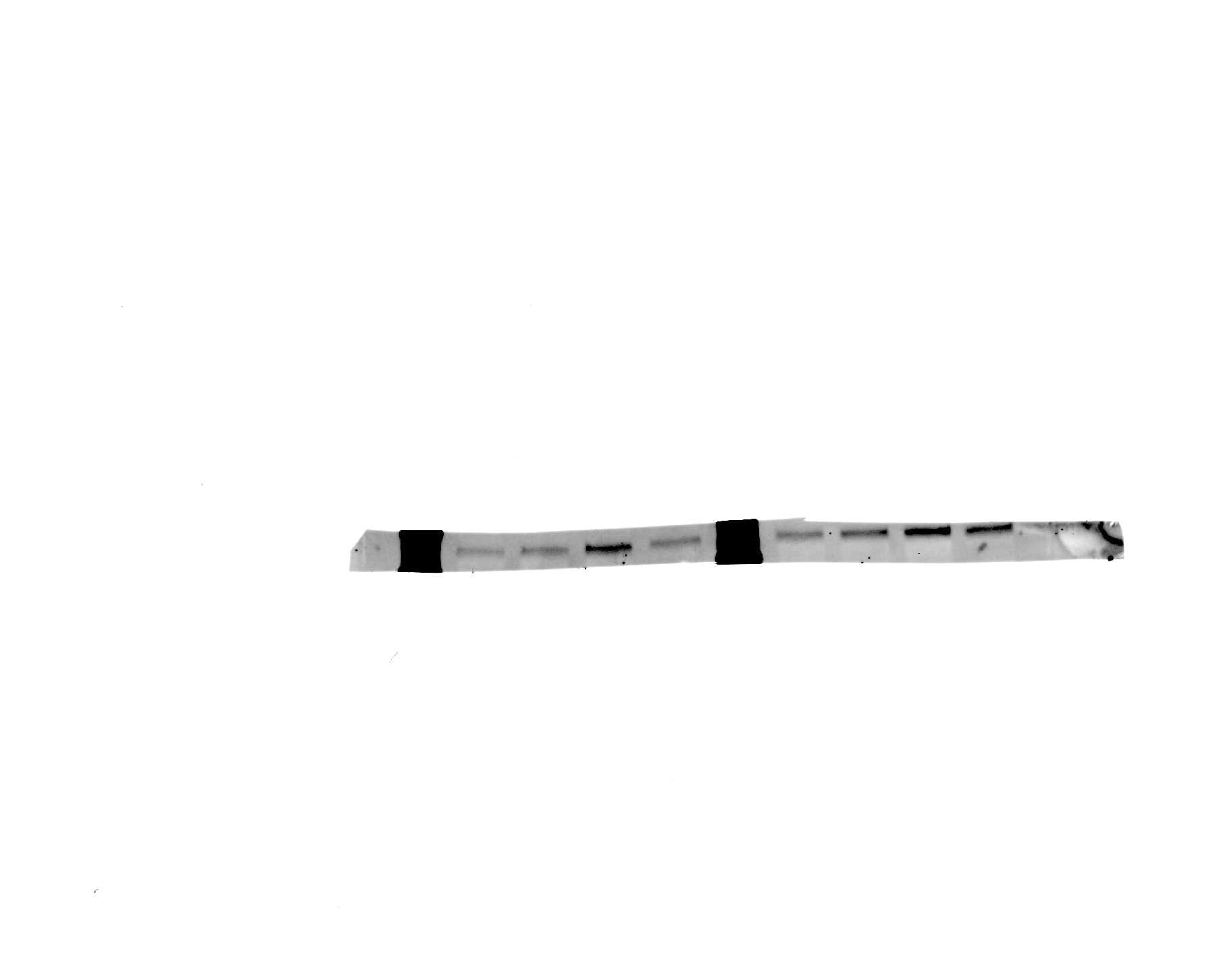

Supplement: Figure 3—source data 2. [file elife-102980-fig3-data2.zip › Figure 3 - source data 2/pAMPK.tif]

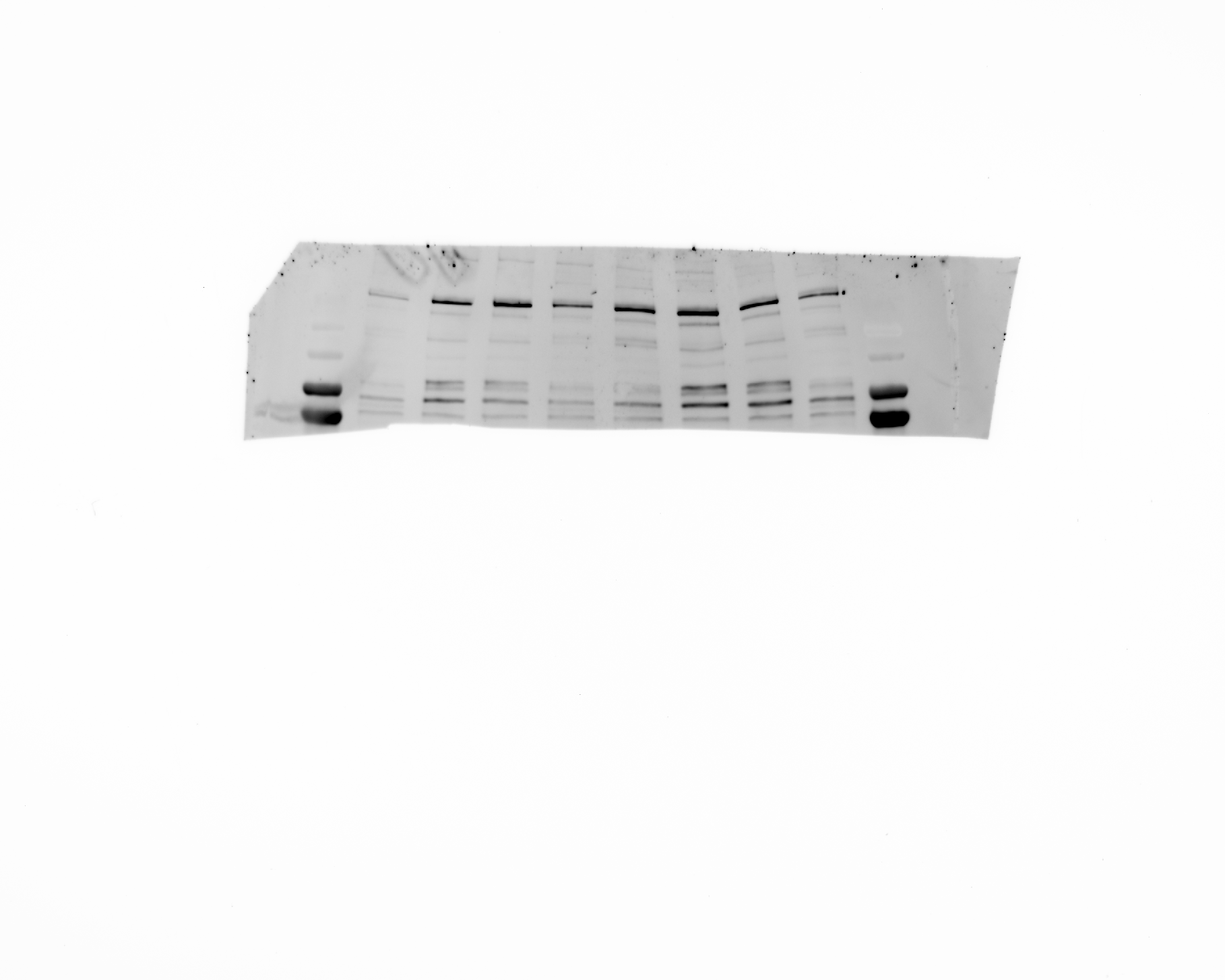

Supplement: Figure 3—source data 2. [file elife-102980-fig3-data2.zip › Figure 3 - source data 2/HIF1a .tif]

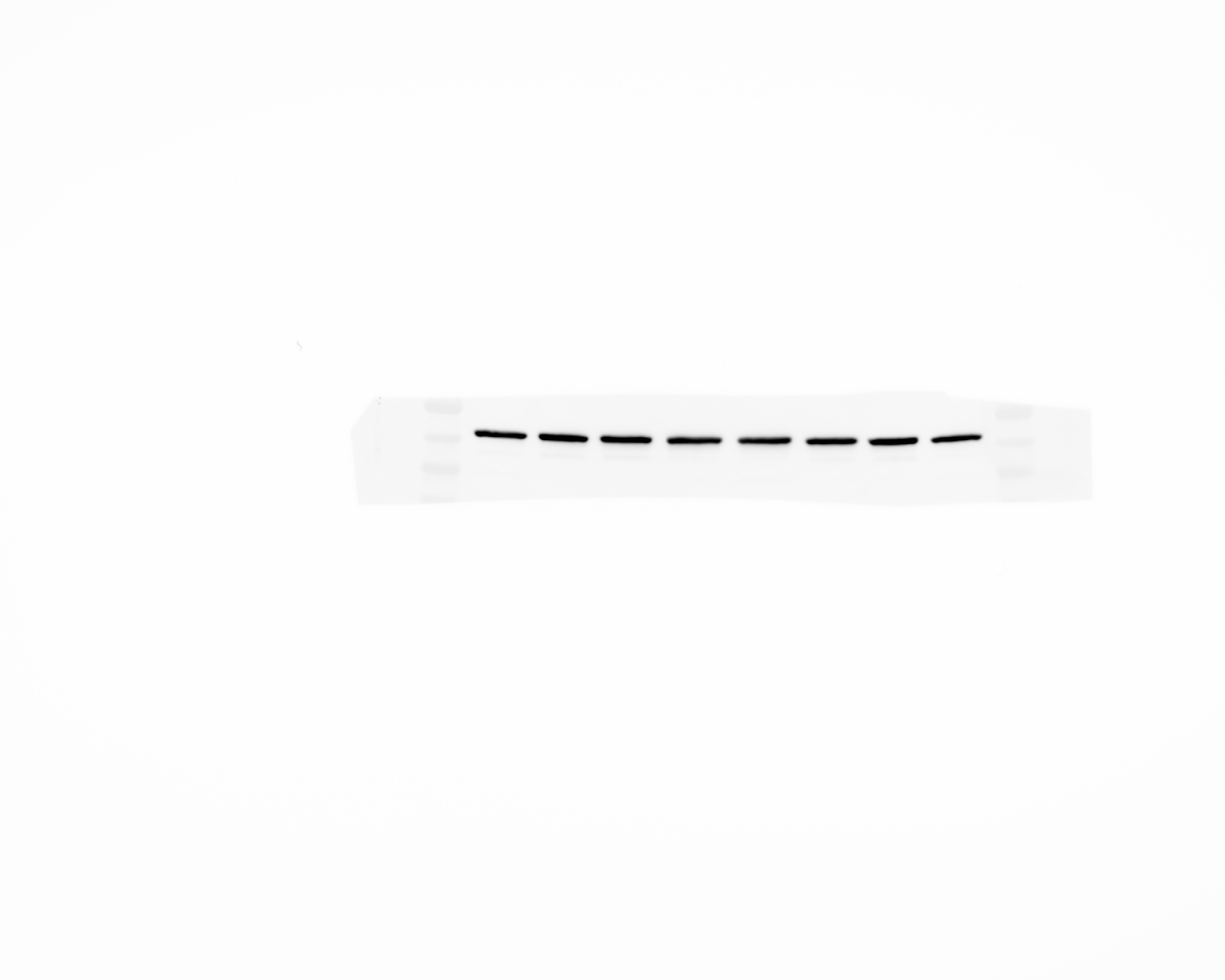

Supplement: Figure 3—source data 2. [file elife-102980-fig3-data2.zip › Figure 3 - source data 2/bActin1_HIF1a.tif]

Figure 3 - figure supplement 1A

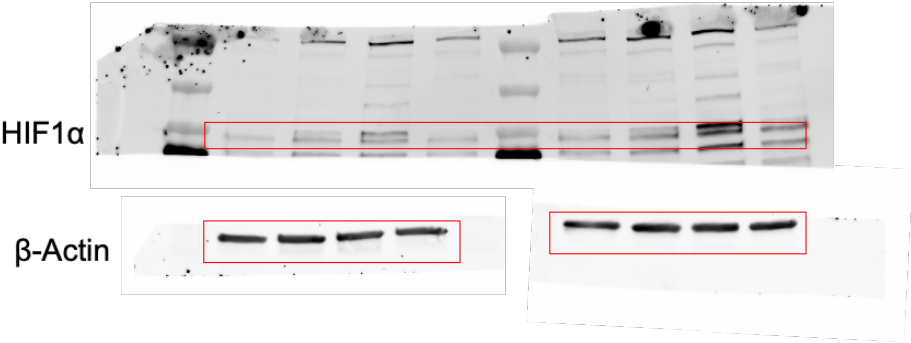

Figure 3 - figure supplement 1B

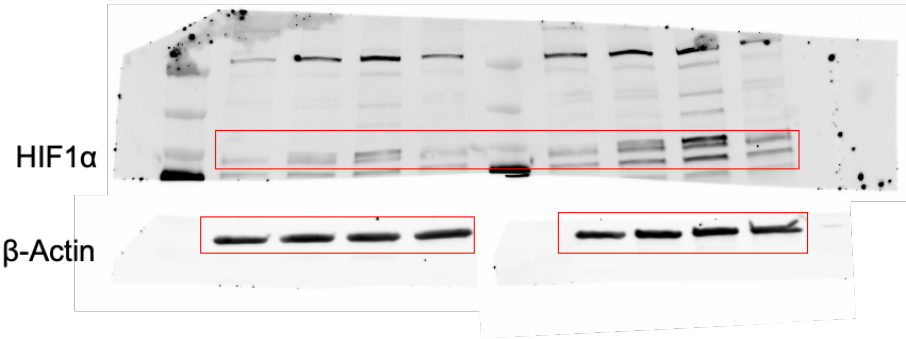

Figure 3 - figure supplement 1C

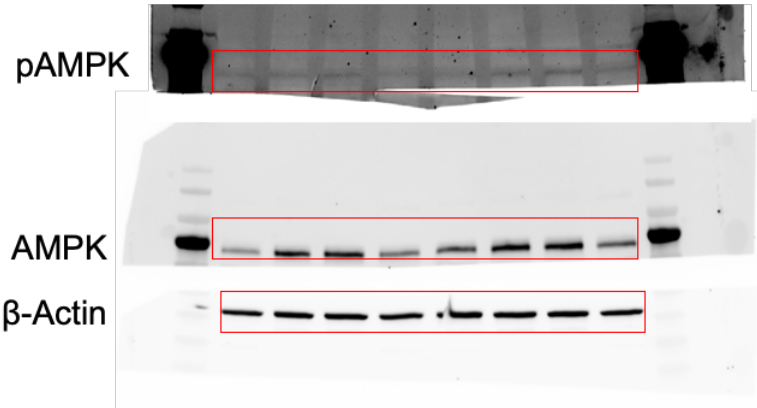

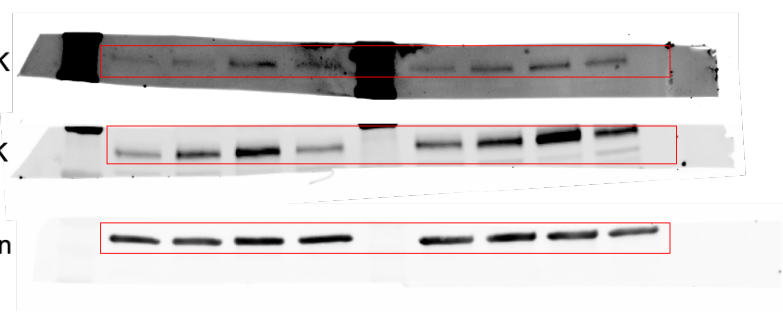

Supplement: Figure 3—figure supplement 1—source data 1. [file elife-102980-fig3-figsupp1-data1.pdf]

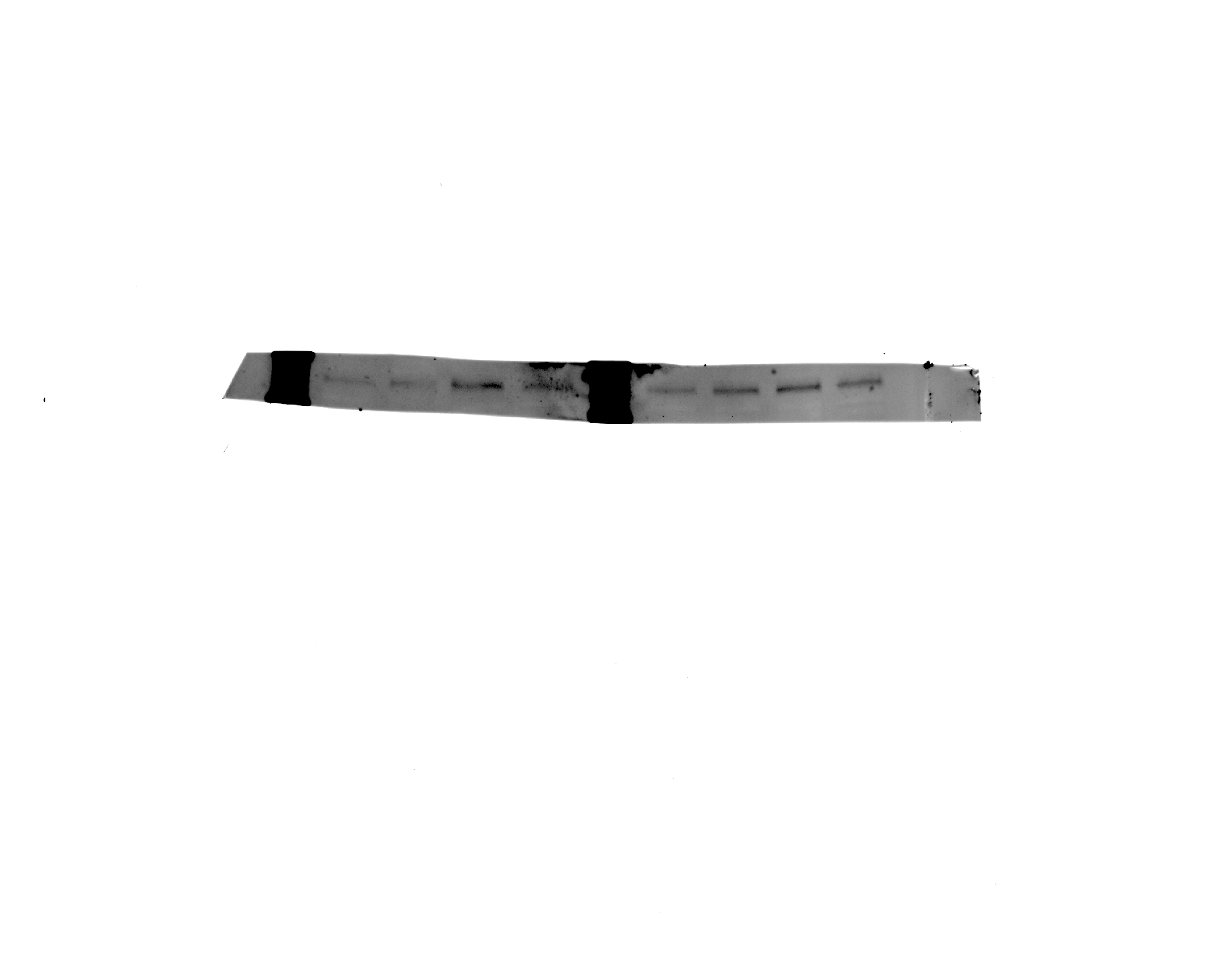

Supplement: Figure 3—figure supplement 1—source data 2. [file elife-102980-fig3-figsupp1-data2.zip › Figure 3 - figure supplement 1 - source data 2/pAMPK_1D.tif]

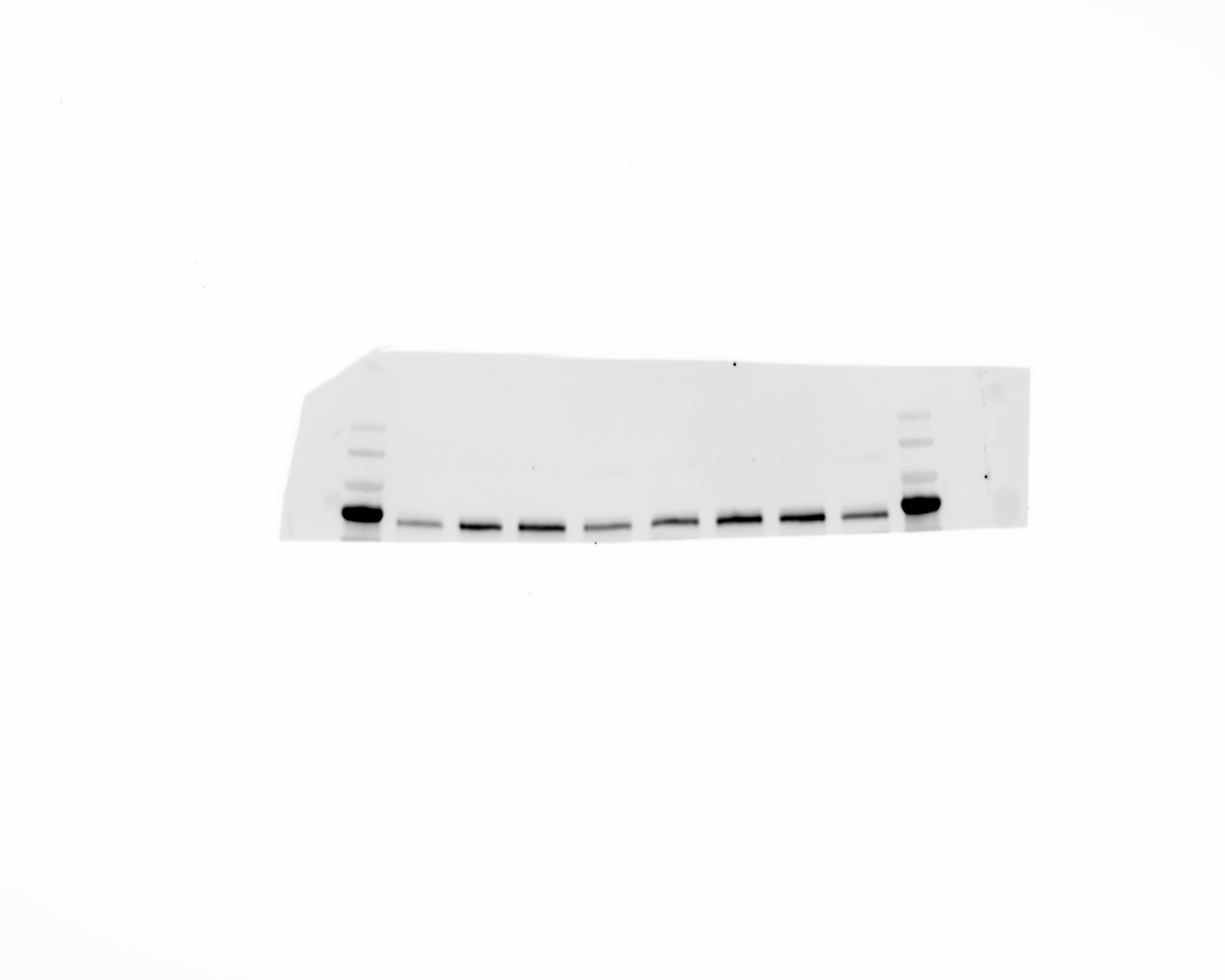

Supplement: Figure 3—figure supplement 1—source data 2. [file elife-102980-fig3-figsupp1-data2.zip › Figure 3 - figure supplement 1 - source data 2/AMPK_1C.tif]

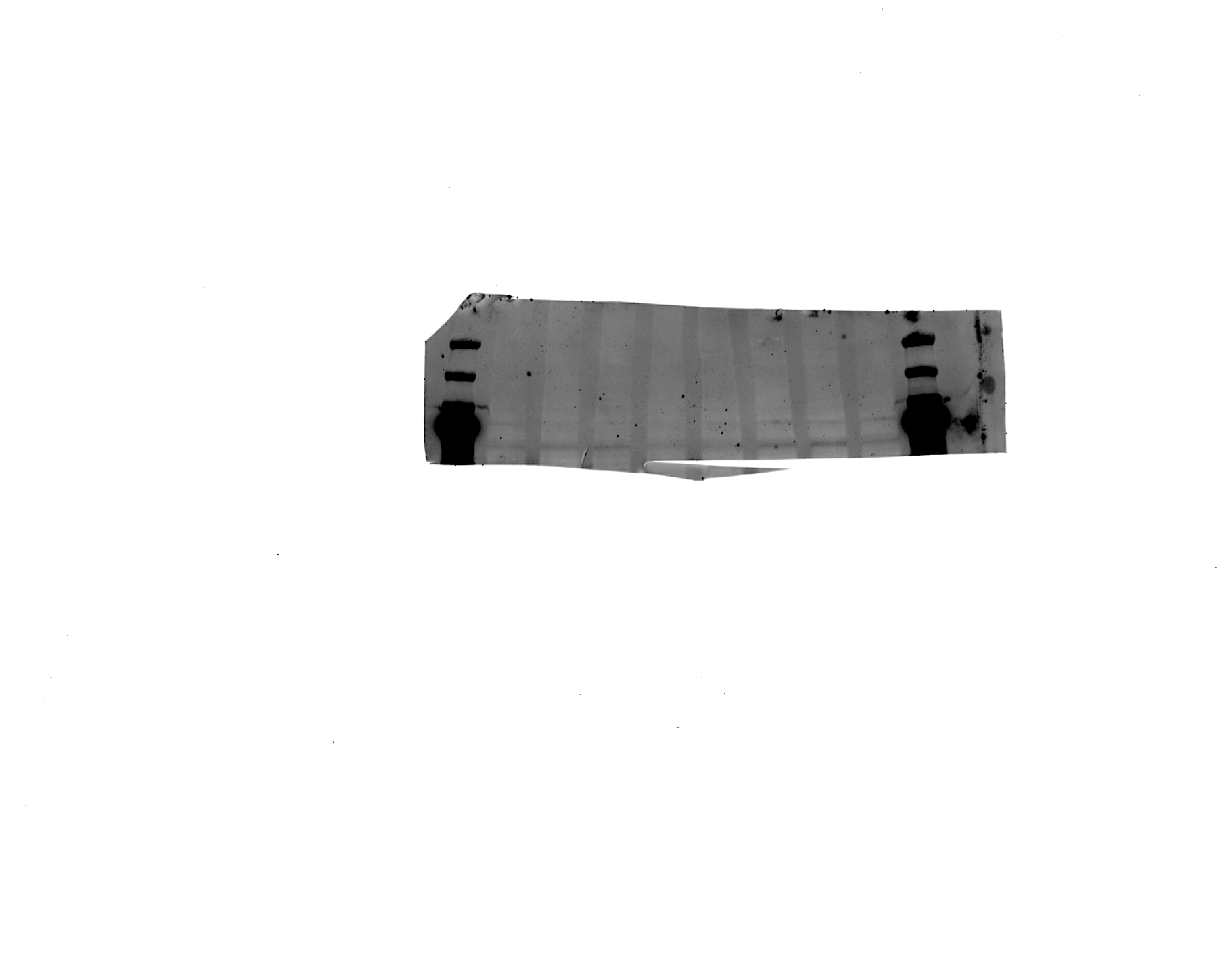

Supplement: Figure 3—figure supplement 1—source data 2. [file elife-102980-fig3-figsupp1-data2.zip › Figure 3 - figure supplement 1 - source data 2/pAMPK_1C.tif]

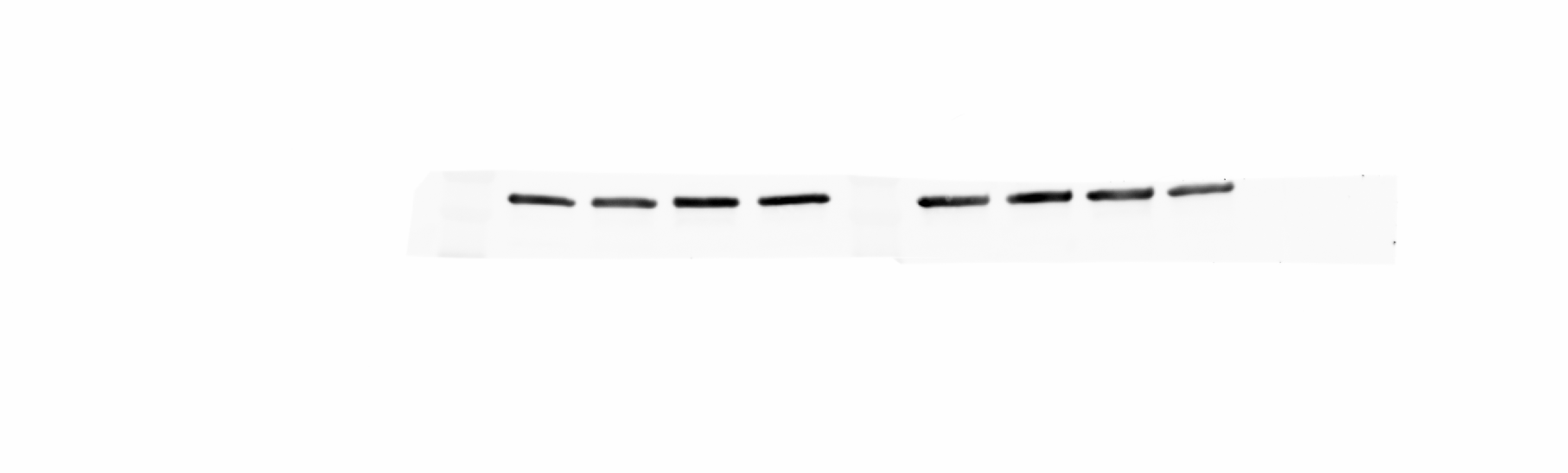

Supplement: Figure 3—figure supplement 1—source data 2. [file elife-102980-fig3-figsupp1-data2.zip › Figure 3 - figure supplement 1 - source data 2/bActin4_AMPK_1D.tif]

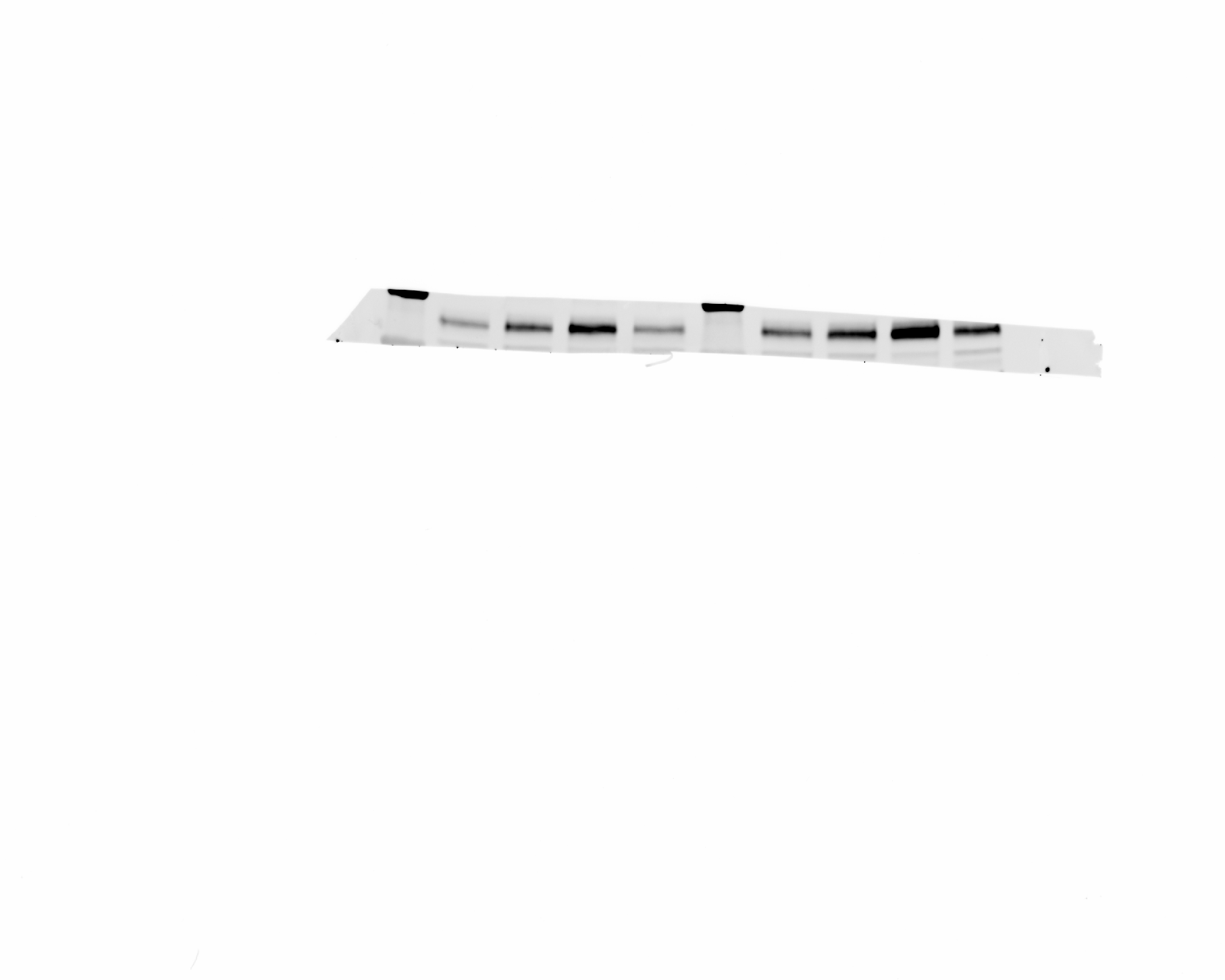

Supplement: Figure 3—figure supplement 1—source data 2. [file elife-102980-fig3-figsupp1-data2.zip › Figure 3 - figure supplement 1 - source data 2/AMPK_1D.tif]

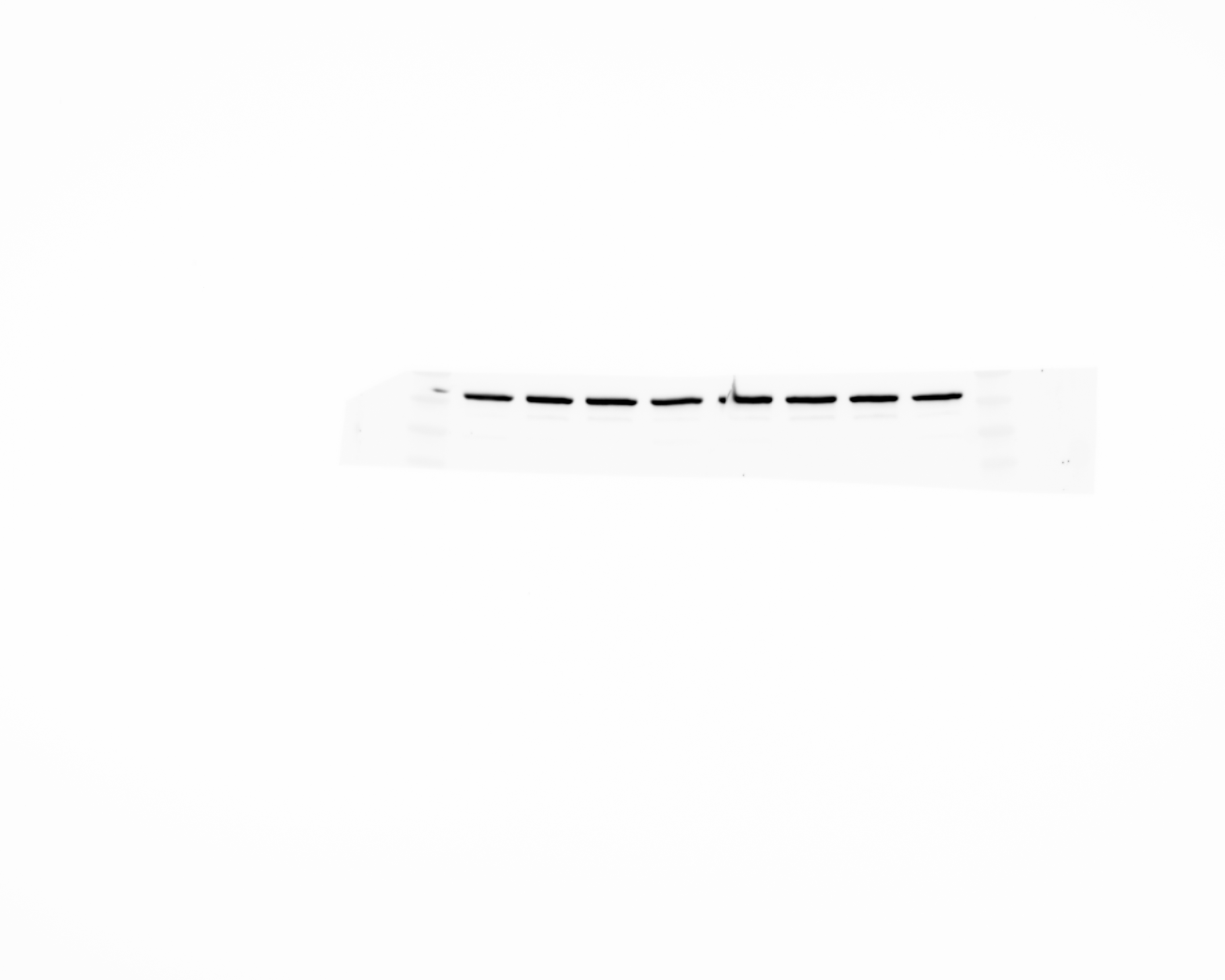

Supplement: Figure 3—figure supplement 1—source data 2. [file elife-102980-fig3-figsupp1-data2.zip › Figure 3 - figure supplement 1 - source data 2/bActin3_AMPK_1C.tif]

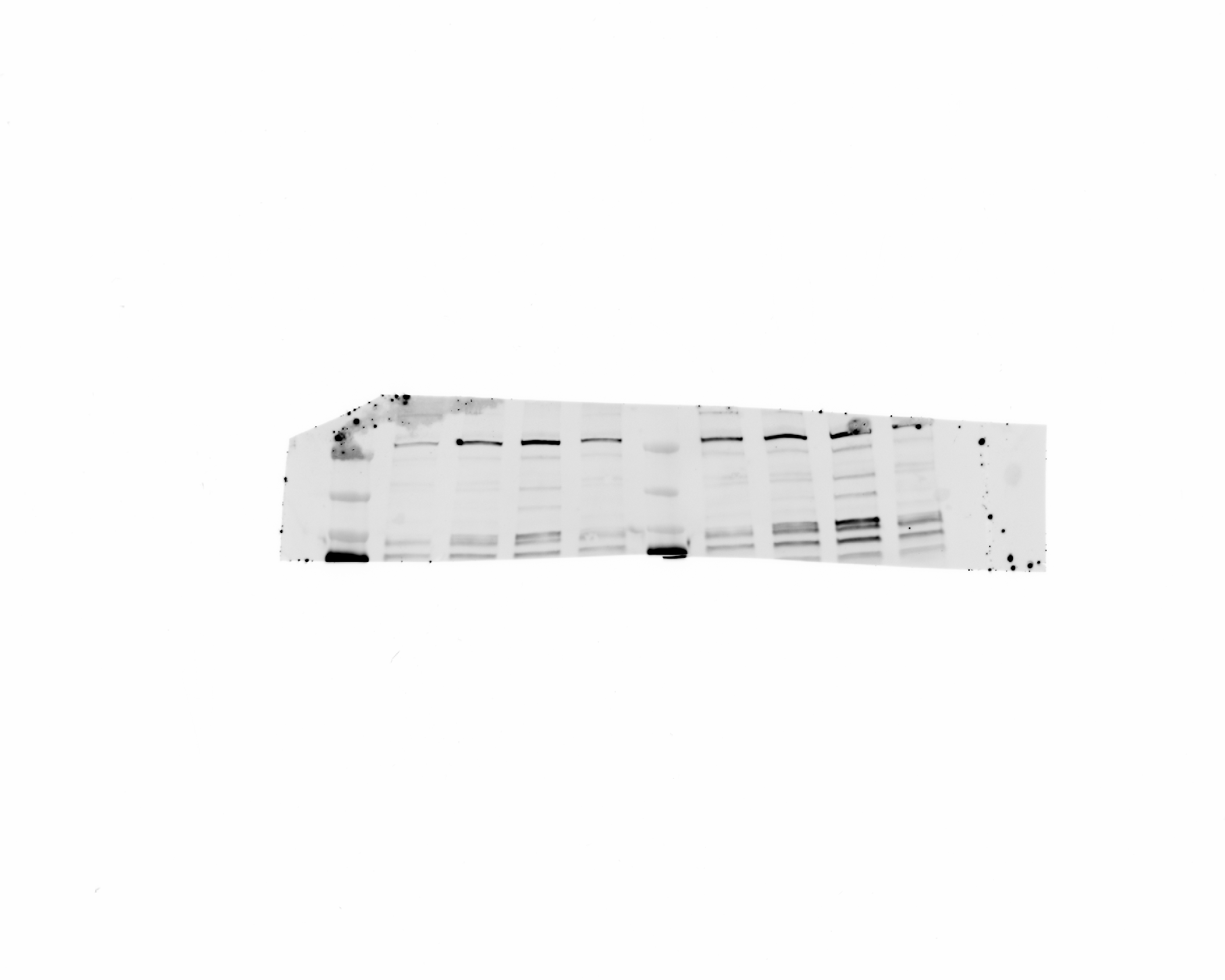

Supplement: Figure 3—figure supplement 1—source data 2. [file elife-102980-fig3-figsupp1-data2.zip › Figure 3 - figure supplement 1 - source data 2/HIF1a_1B.tif]

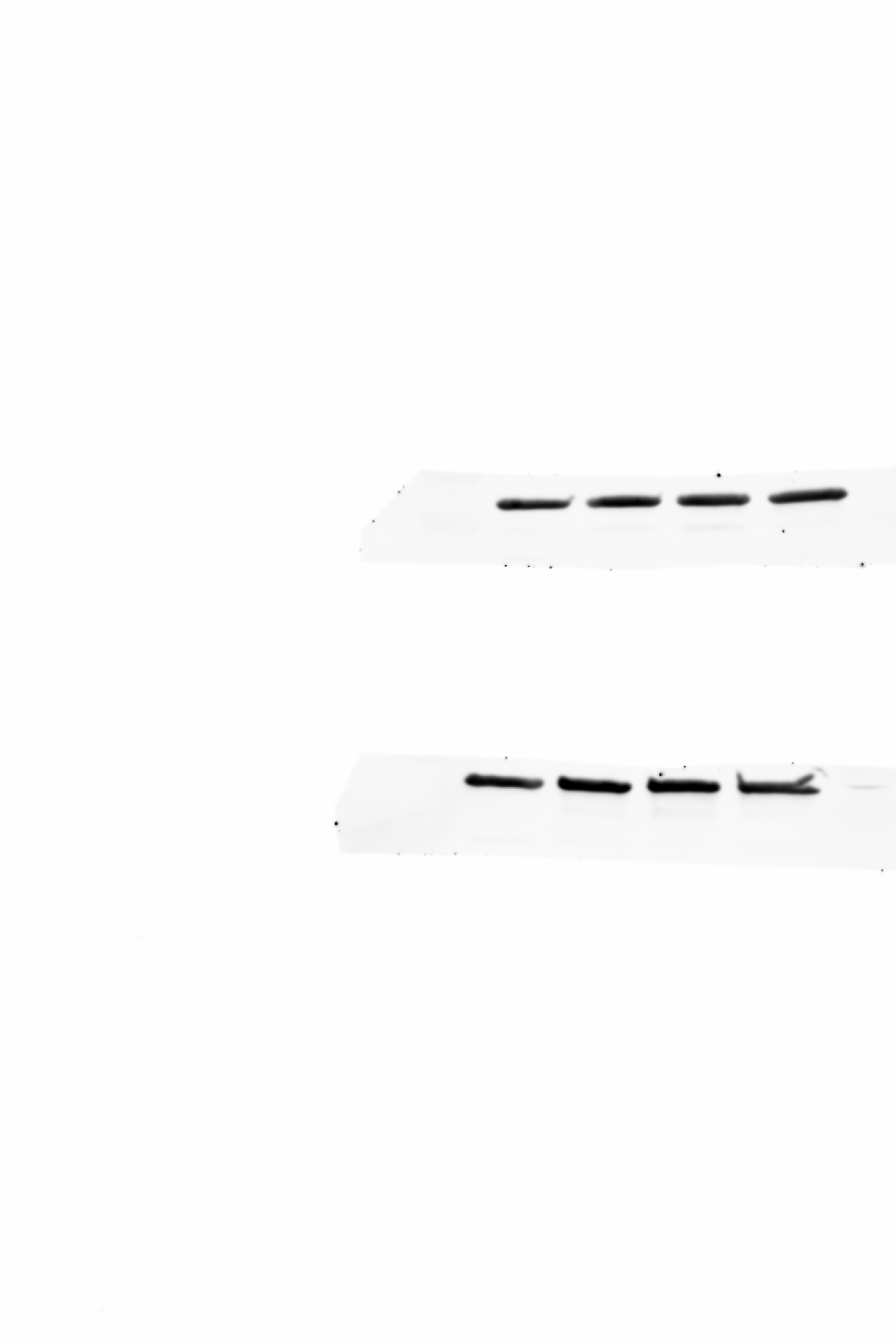

Supplement: Figure 3—figure supplement 1—source data 2. [file elife-102980-fig3-figsupp1-data2.zip › Figure 3 - figure supplement 1 - source data 2/bActin2_HIF1a_1B.tif]

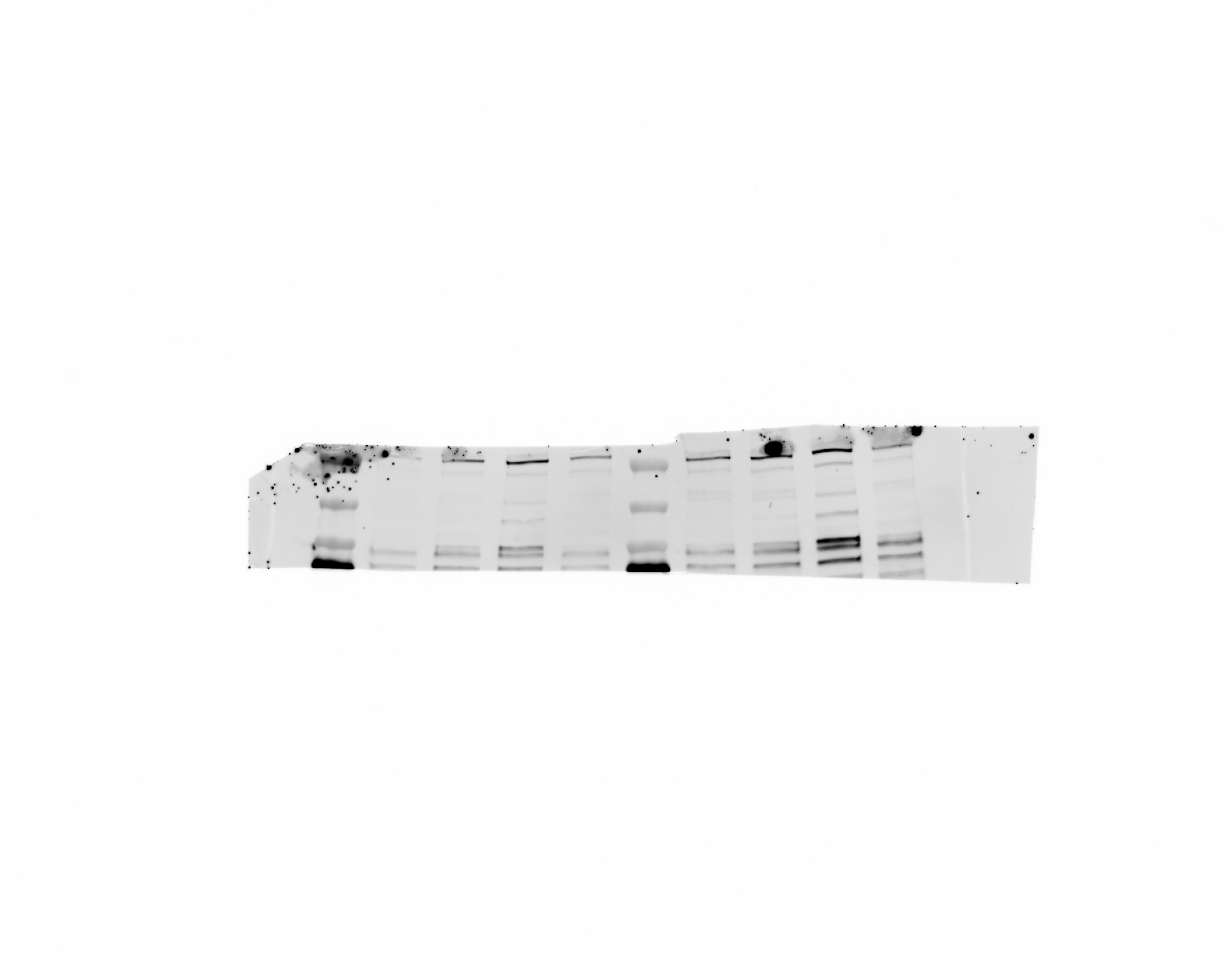

Supplement: Figure 3—figure supplement 1—source data 2. [file elife-102980-fig3-figsupp1-data2.zip › Figure 3 - figure supplement 1 - source data 2/HIF1a_1A.tif]

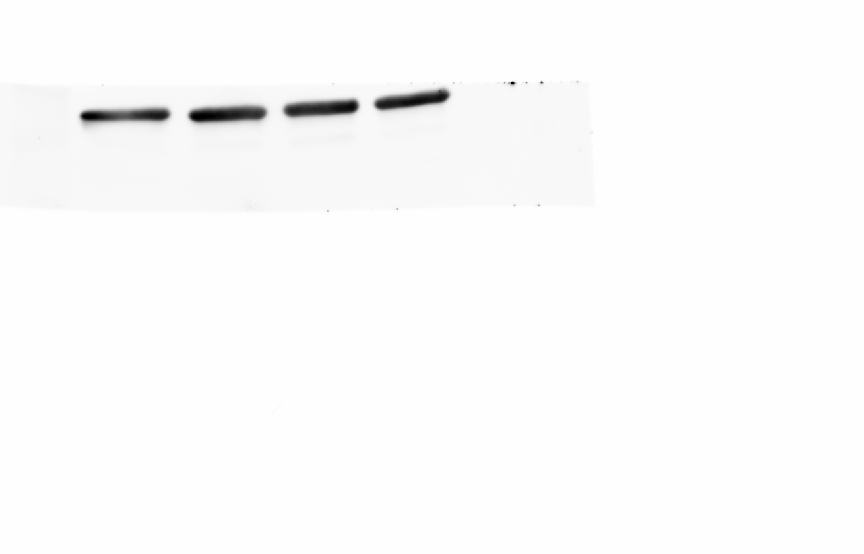

Supplement: Figure 3—figure supplement 1—source data 2. [file elife-102980-fig3-figsupp1-data2.zip › Figure 3 - figure supplement 1 - source data 2/bActin1_HIFa_1A_part2.tif]

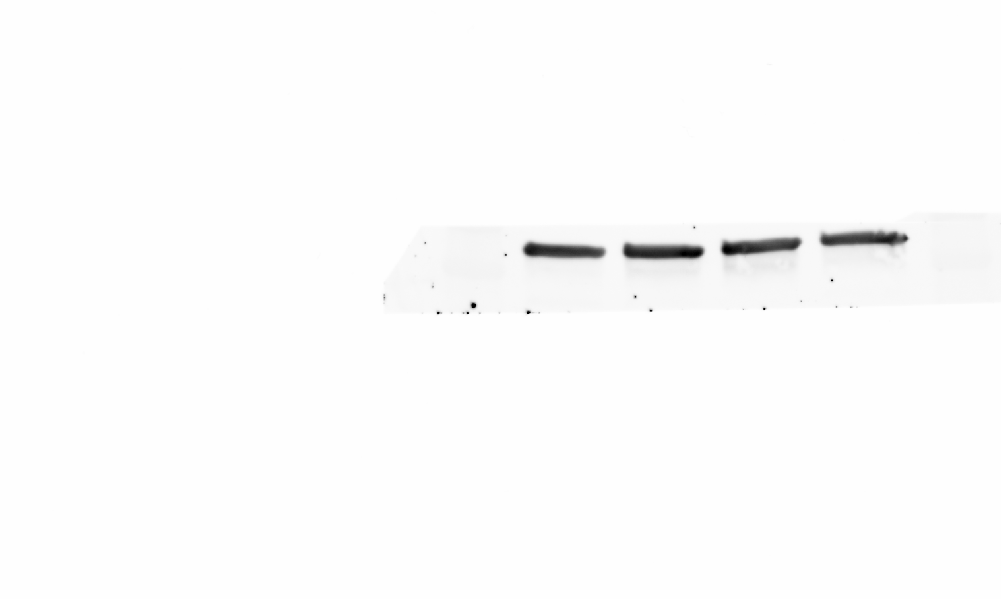

Supplement: Figure 3—figure supplement 1—source data 2. [file elife-102980-fig3-figsupp1-data2.zip › Figure 3 - figure supplement 1 - source data 2/bActin1_HIFa_1A_part1.tif]

Figure 3 - figure supplement 2A

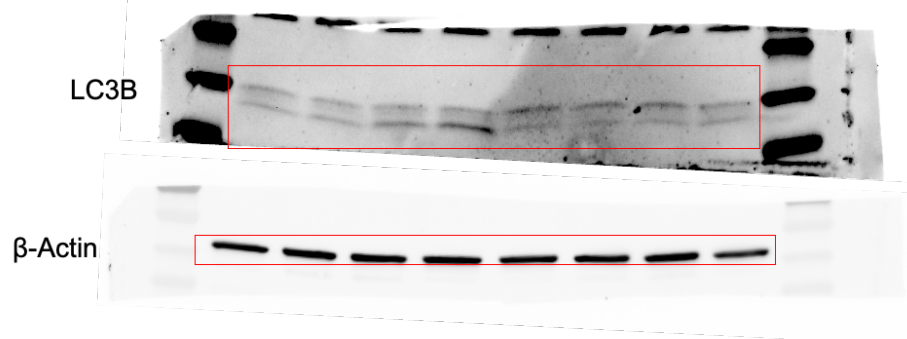

Figure 3 - figure supplement 2B

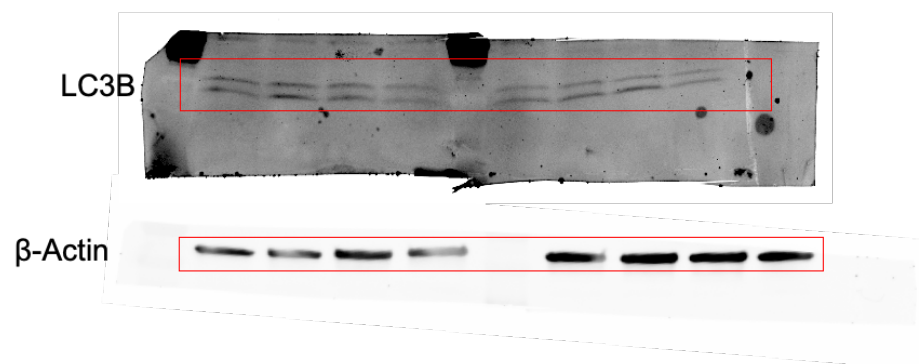

Supplement: Figure 3—figure supplement 2—source data 1. [file elife-102980-fig3-figsupp2-data1.pdf]

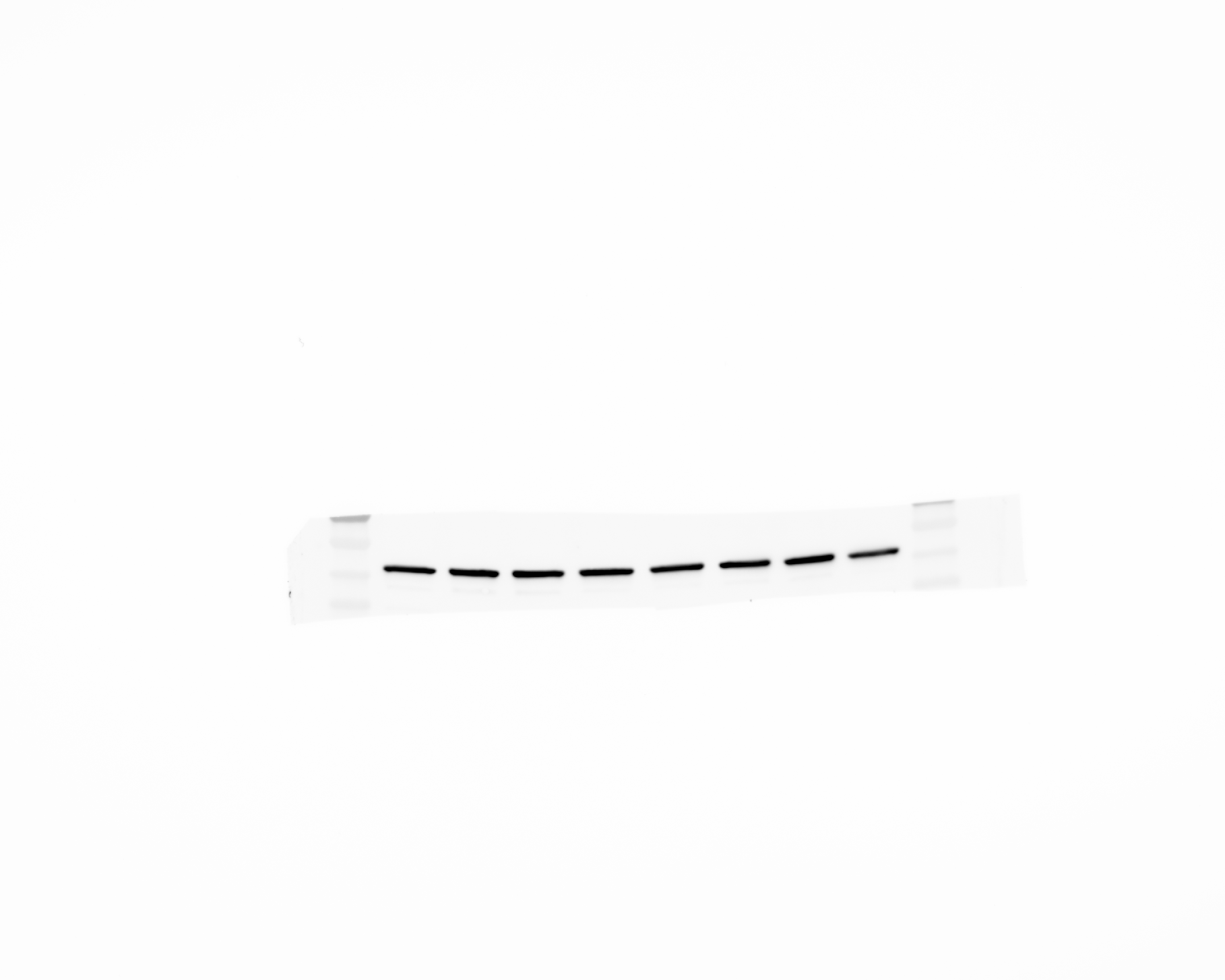

Supplement: Figure 3—figure supplement 2—source data 2. [file elife-102980-fig3-figsupp2-data2.zip › Figure 3 - figure supplement 2 - source data 2/bActin1_LC3B_2A.tif]

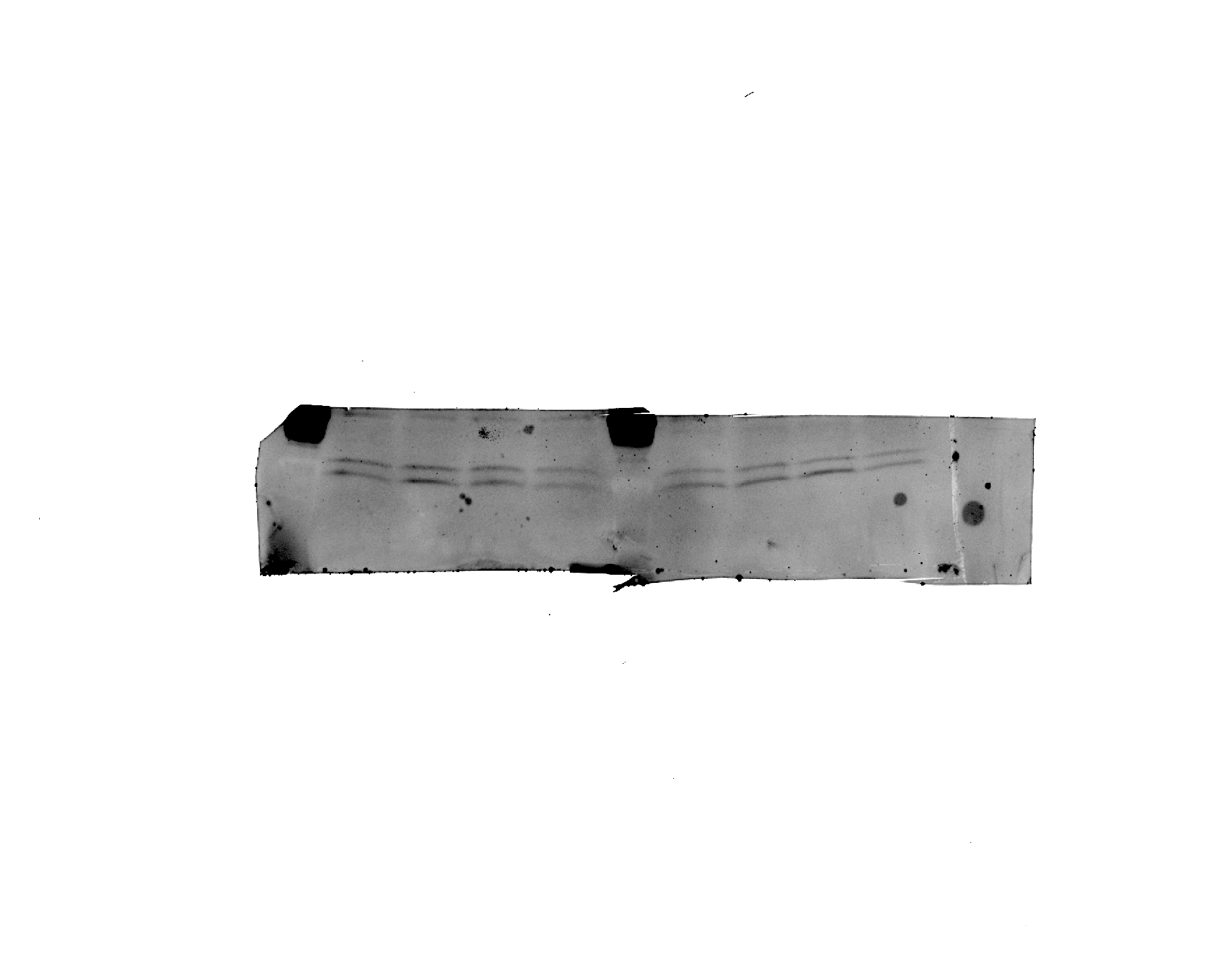

Supplement: Figure 3—figure supplement 2—source data 2. [file elife-102980-fig3-figsupp2-data2.zip › Figure 3 - figure supplement 2 - source data 2/LC3B_2B.tif]

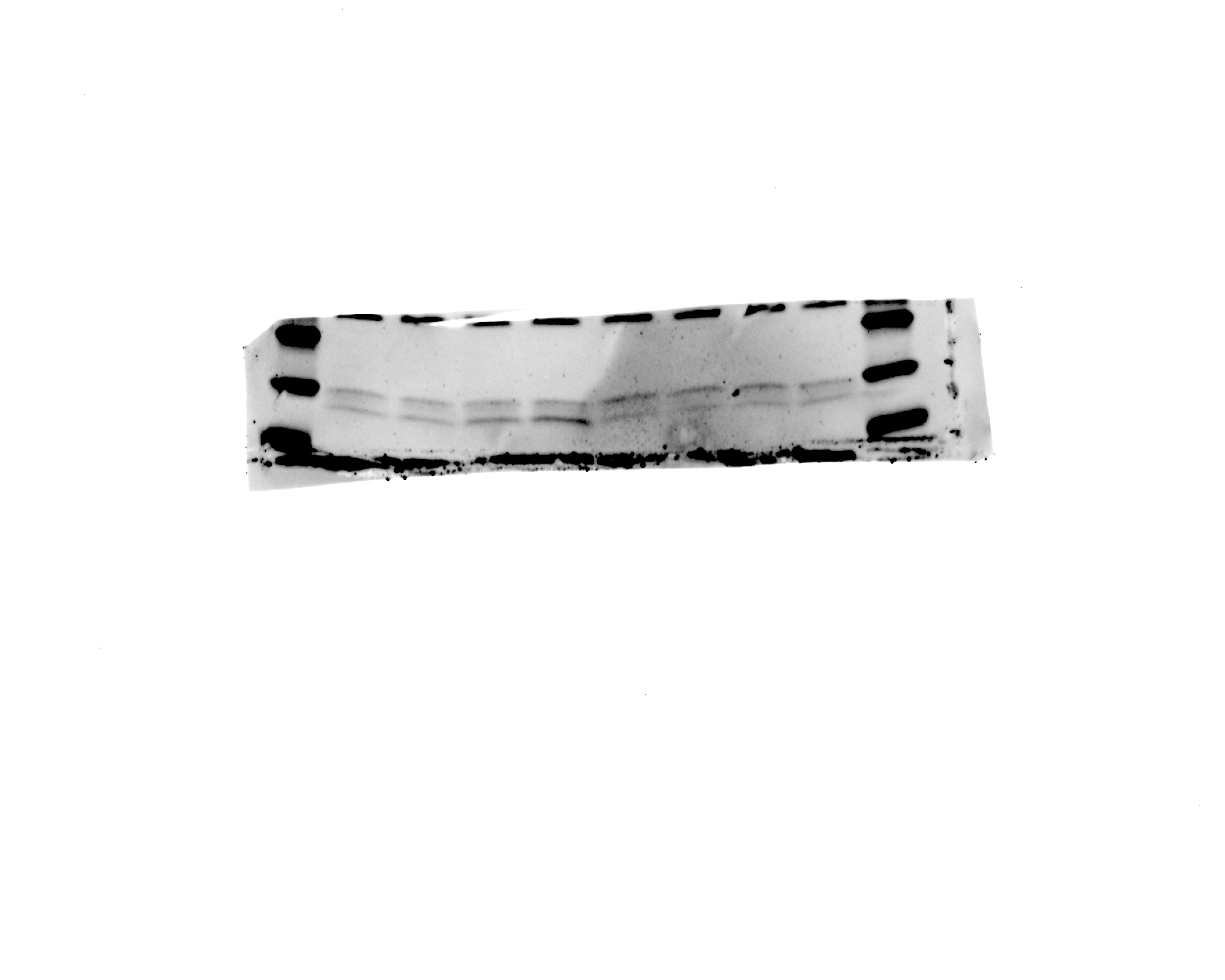

Supplement: Figure 3—figure supplement 2—source data 2. [file elife-102980-fig3-figsupp2-data2.zip › Figure 3 - figure supplement 2 - source data 2/LC3B_2A.tif]

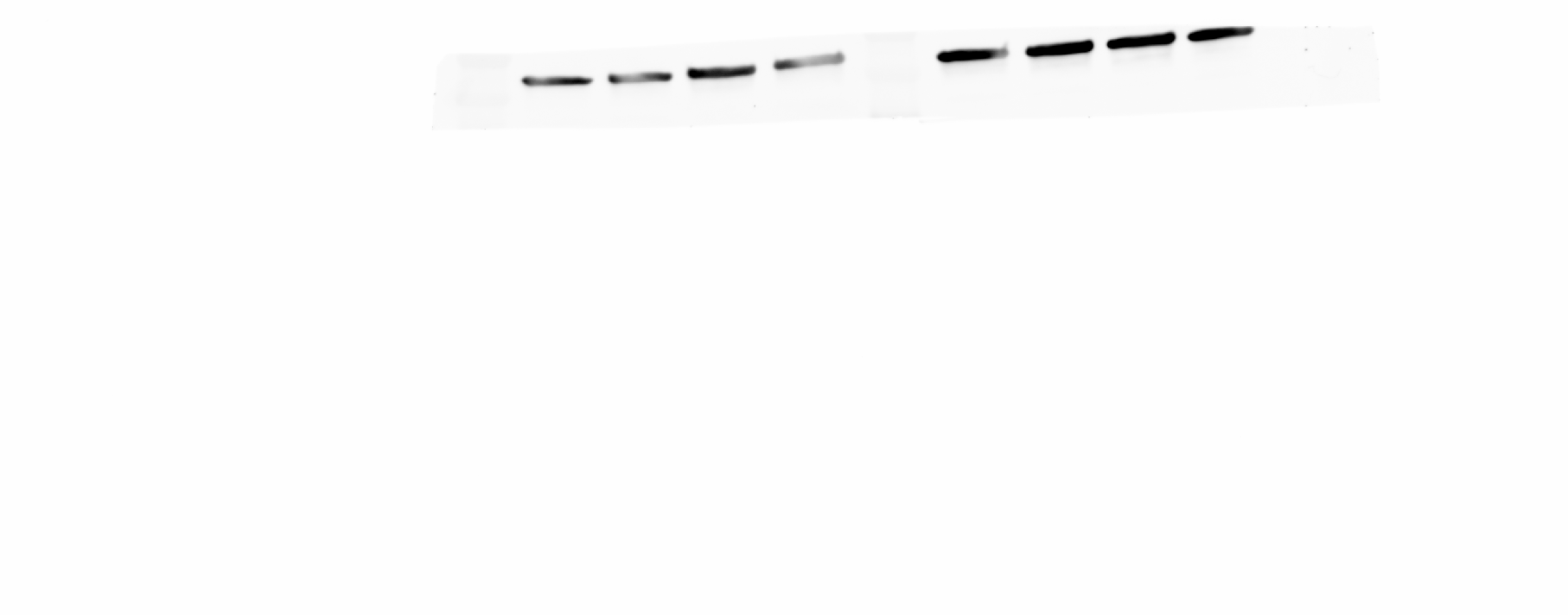

Supplement: Figure 3—figure supplement 2—source data 2. [file elife-102980-fig3-figsupp2-data2.zip › Figure 3 - figure supplement 2 - source data 2/bActin2_LC3B_2B.tif]
